# *Acinetobacter nosocomialis* utilizes a unique type VI secretion system to promote its survival in niches with prey bacteria

**DOI:** 10.1128/mbio.01468-24

**Published:** 2024-06-25

**Authors:** Yu Sun, Lidong Wang, Ming Zhang, Jing Jie, Qingtian Guan, Jiaqi Fu, Xiao Chu, Dong Chen, Chunxiuli Li, Lei Song, Zhao-Qing Luo

**Affiliations:** 1 Department of Respiratory Medicine, Center of Infectious Diseases and Pathogen Biology, Key Laboratory of Organ Regeneration and Transplantation of the Ministry of Education, State Key Laboratory for Diagnosis and Treatment of Severe Zoonotic Infectious Diseases, The First Hospital of Jilin University, Changchun, China; 2 Department of Gastroenterology, Endoscopy center, The First Hospital of Jilin University, Changchun, China; 3 Department of Ultrasound, The First Hospital of Jilin University, Changchun, China; 4 Bioinformatics Laboratory, The First Hospital of Jilin University, Changchun, China; 5 Department of Biological Sciences, Purdue University, West Lafayette, Indiana, USA; University of Illinois Chicago, Chicago, Illinois, USA

**Keywords:** bacterial competition, effectors, RHS toxins, DNase

## Abstract

Pathogenic bacteria of the *Acinetobacter* genus pose a severe threat to human health worldwide due to their strong adaptability, tolerance, and antibiotic resistance. Most isolates of these bacteria harbor a type VI secretion system (T6SS) that allows them to outcompete co-residing microorganisms, but whether this system is involved in acquiring nutrients from preys remains less studied. In this study, we found that Ab25, a clinical isolate of *Acinetobacter nosocomialis,* utilizes a T6SS to kill taxonomically diverse microorganisms, including bacteria and fungi. The T6SS of Ab25 is constitutively expressed, and among the three predicted effectors, T6e1, a member of the RHS effector family, contributes the most for its antimicrobial activity. T6e1 undergoes self-cleavage, and a short carboxyl fragment with nuclease activity is sufficient to kill target cells via T6SS injection. Interestingly, strain Ab25 encodes an orphan VgrG protein, which when overexpressed blocks the firing of its T6SS. In niches such as dry plastic surfaces, the T6SS promotes prey microorganism-dependent survival of Ab25. These results reveal that *A. nosocomialis* employs T6SSs that are highly diverse in their regulation and effector composition to gain a competitive advantage in environments with scarce nutrient supply and competing microbes.

**IMPORTANCE:**

The type VI secretion system (T6SS) plays an important role in bacterial adaptation to environmental challenges. Members of the *Acinetobacter* genus, particularly *A. baumannii* and *A. nosocomialis,* are notorious for their multidrug resistance and their ability to survive in harsh environments. In contrast to *A. baumannii*, whose T6SS has been well-studied, few research works have focused on *A. nosocomialis*. In this study, we found that an *A. nosocomialis* strain utilizes a contitutively active T6SS to kill diverse microorganisms, including bacteria and fungi. Although T6SS structural proteins of *A. nosocomialis* are similar to those of *A. baumannii*, the effector repertoire differs greatly. Interestingly, the T6SS of the *A. nosocomialis* strain codes for an ophan VgrG protein, which blocks the firing of the system when overexpressed, suggesting the existence of a new regulatory mechanism for the T6SS. Importantly, although the T6SS does not provide an advantage when the bacterium is grown in nutrient-rich medium, it allows *A. nosocomialis* to survive better in dry surfaces that contain co-existing bacteria. Our results suggest that killing of co-residing microorganisms may increase the effectiveness of strategies designed to reduce the fitness of *Acinetobacter* bacteria by targeting their T6SS.

## INTRODUCTION

Members of the *Acinetobacter* genus, especially *A. baumannii*, *A. nosocomialis,* and *A. pittii* are widely recognized as important etiological agents responsible for healthcare facility-associated infections (1). These pathogens cause infections in various parts of the human body, including the respiratory system, the urinary tract, and bloodstream (2–4). Infections by *Acinetobacter* spp. are particularly common in hospital settings and among immunocompromised individuals. One reason for the high prevalence of infections by these bacteria is their ability to adapt to various environmental challenges, including low nutrient availability, dryness, and temperature fluctuations (5, 6); these traits enable them to survive and grow in conditions unfavorable to growth of other microorganisms (7–9). Medical devices are common colonization sites for *Acinetobacter* bacteria in healthcare settings (10), where the bacteria can survive for extended periods and have the potential to cause infections. Another noted feature associated with these bacteria is their resistance to a wide spectrum of antibiotics, including broad-spectrum β-lactams and aminoglycosides (11). In recent years, there has been a significant increase in multidrug-resistant strains of *Acinetobacter* (12). These strains present a grave challenge to clinicians due to limited therapeutic options, which has made infection management more complicated.

Specialized protein secretion systems play important roles in virulence, interspecies competition, and exchange of genetic materials such as genes for drug resistance (13). Among these, the type VI secretion system (T6SS) arguably is the most widely distributed bacterial injection machinery, and some bacterial isolates harbor multiple systems, with each being dedicated to a distinct function (14). T6SS-mediated antagonistic interactions are important for infection or colonization of specific niches by bacteria in various scenarios. For instance, during intestinal infections, pathogens utilize the T6SS to overcome colonization barriers elaborated by resident microbiota (15). Some bacteria employ the T6SS to evade attacks from the host immune system (16, 17). The T6SS has also been shown to promote horizontal gene transfer (18) or to maintain stable symbiotic relationships in complex environments (19). Under conditions such as oxidative stress, some bacteria use metal ion-binding T6SS effectors to acquire essential nutrients for their survival (20–22).

The T6SS nanomachine consists of an extracellular appendage and associated complexes formed by multiple proteins, including extracellular sheath assembly proteins, membrane-anchored components, sheath tube components, and effector proteins (23). Effector proteins are the primary executioner molecules released by the T6SS. In most cases, sensing competing bacteria by direct contact activates the T6SS, resulting in the injection of effectors into target cells (24). Although some T6SS effectors have been shown to target proteins in eukaryotic hosts (25, 26), the majority of the known effectors function against competing microorganisms, including Gram-negative, -positive bacteria, and fungi (27–29). These effectors employ a range of mechanisms to kill the target cells, including membrane lysis (30), depleting key metabolites (31), and attacking genetic materials such as DNA (32).

The T6SS has been identified in many *A. baumannii* isolates (33–35). In the prototype strain ATCC17978, the expression of the T6SS is repressed by two TetR-like proteins harbored by its large plasmid pAB3 (36). Because pAB3 is a conjugative plasmid harboring multiple drug resistance genes (37), it is believed that such repression allows the spread of resistance genes (36). Alternatively, repression may prevent the killing of kin bacteria that could potentially serve as the recipient of the plasmid. Once derepressed, *A. baumannii* uses the T6SS to kill bacteria and fungi with effectors such as Tse4 (27, 28) and TafE (38) with peptidoglycanase and DNase activity, respectively.

To further study the function of the T6SS in *Acinetobacter* spp., we screened a collection of clinical isolates and found that an *A. nosocomialis* strain displayed robust antibacterial activity by a constitutively expressed T6SS. Further study reveals that this T6SS aids bacterial survival in nutrient-scarce niches with prey microorganisms and that its firing appears to be regulated by an orphan VgrG protein.

## RESULTS

### The clinical *Acinetobacter* strain Ab25 harbors a functional T6SS

**Fig 1:**
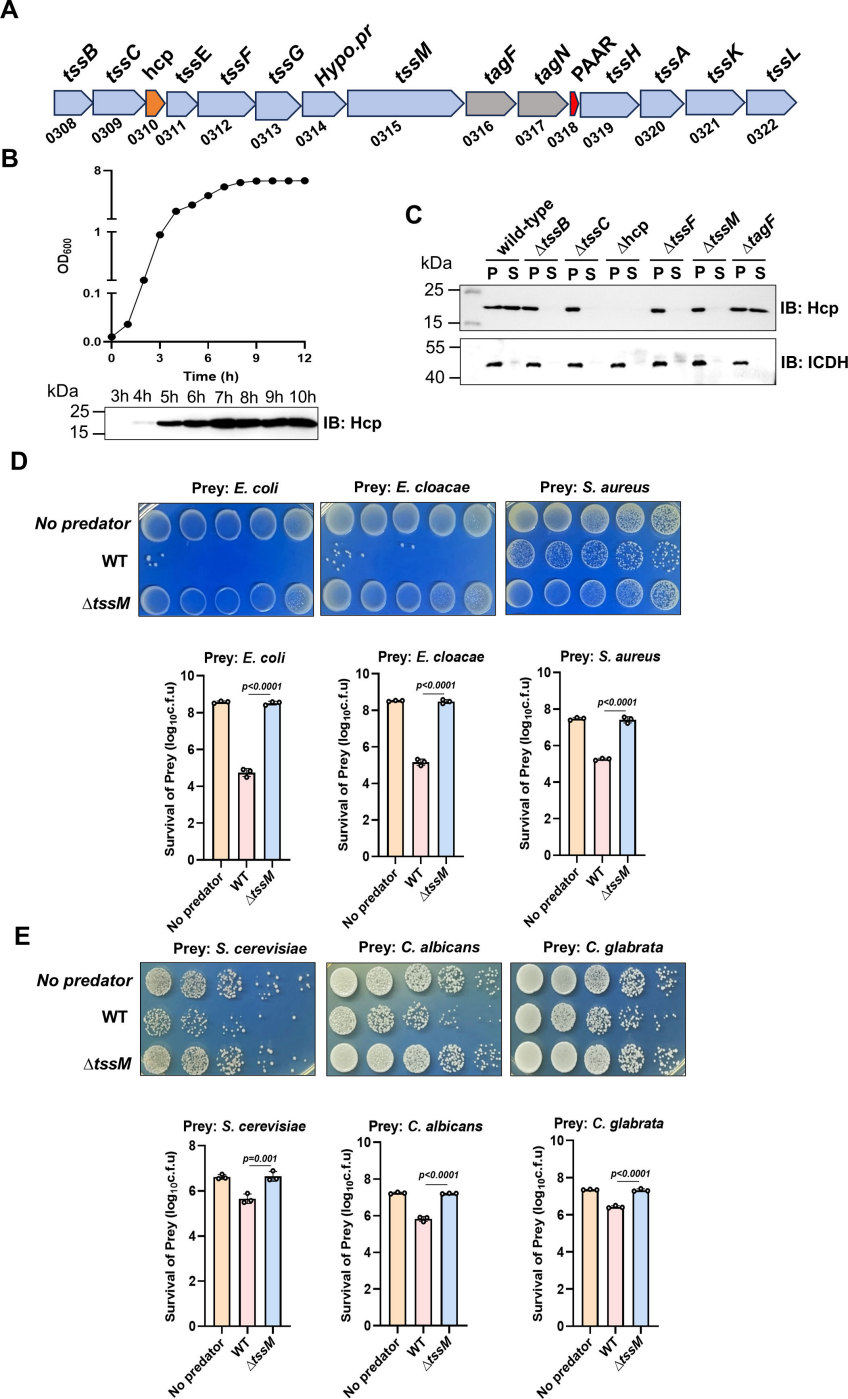
*A*. *nosocomialis* strain Ab25 harbors a functional T6SS (**A)** A diagram of the gene cluster that harbors T6SS component genes in *A. nosocomialis* strain Ab25. (**B)** Growth phase-dependent expression of Hcp and its secretion. Saturated cultures were diluted 1:500 (OD_600_ = 0.01), and bacterial growth was monitored by measuring OD_600_ (upper). Samples withdrawn at the indicated timepoints were detected for Hcp by immunoblotting (lower). Note that the Hcp protein only became detectable after 4 hours of growth (OD_600_≈2.0). (**C)** The T6SS is required for Hcp secretion. The culture supernatant and total cell lysates of the indicated bacterial strains were probed for Hcp by immunoblotting. The cytosolic isocitrate dehydrogenase (ICDH) was detected as a control. (**D)** Killing of bacteria by strain Ab25. Cells of wild-type or the Δ*tssM* mutant were mixed with cells of the indicated bacteria at a 1:1 ratio. After incubation for 4 hours, the survival of the prey bacteria was determined by spotting diluted cells onto selective media. Results shown were representative images of the plating (upper panels) and quantitative assessment of the survival rates (lower panels). (**E)** Antifungal activity of strain Ab25. Bacteria of wild-type or the Δ*tssM* mutant were mixed with the indicated fungi at a ratio of 10:1. After incubation for 6 hours, the survival of the fungal cells was determined by serial dilutions and plating to count total colony-forming units (CFUs). Images of representative experiments and quantitative results are shown (upper panels). In panels D and E, results (mean ± S.E.) were from three independent experiments, each done in triplicate.

To probe the ability of clinical isolates of *Acinetobacter* to kill other bacteria, we screened a total of 78 strains collected by the First Hospital of Jilin University over a period of 2 years. These efforts led to the identification of a number of strains capable of effectively killing bacteria such as *Escherichia coli* (Fig. S1A). Further tests revealed that strain Ab25 has the most robust antimicrobial activity, and it can target several fungi (see below). To understand its antimicrobial mechanisms, we determined its genome sequence (Fig. S1B). These analyses revealed that this isolate belongs to *A. nosocomialis*. Analysis of its genome sequence revealed that strain Ab25 has a chromosome of 4,042,470 bp, which is predicted to code for 3,970 proteins (Fig. S1B). The genome includes one plasmid of 110,808 bp. We identified a gene cluster that codes for all components of a T6SS (Fig. 1A); these proteins exhibit high-level similarity with their counterparts found in *A. baumannii,* such as strain 17978, with an e value close to 0 for most of the proteins (Table S1).

In some *A. baumannii* strains such as strain 17978, the expression of the T6SS is repressed by TetR-like repressors encoded by large conjugative plasmids (36). We first examined the expression and secretion of Hcp to determine whether the T6SS in strain Ab25 is active under normal conditions. A small amount of Hcp was detected in the culture supernatant of bacteria grown at the exponential phase, and it significantly increased when bacteria entered the stationary phase (Fig. 1B). These results are consistent with the fact that TetR-like proteins capable of inhibiting T6SS expression (36) were absent on the plasmid harbored by strain Ab25. We next determined whether such secretion required a functional T6SS by generating a set of mutants, each lacking a specific predicted T6SS component gene. Hcp secretion no longer occurred in each of these mutants (Fig. 1C). These results indicate that these genes are essential for the function of the T6SS in Ab25.

To further examine the antibacterial activity of the T6SS in Ab25, we tested its ability to affect the survival of several taxonomically distinct microorganisms. Coincubation of Ab25 with *E. coli* or *Enterobacter cloacae* for 4 hours led to approximately 2 orders of magnitude loss in viable cells (Fig. 1D). For the Gram-positive bacterium *Staphylococcus aureus,* such coincubation led to similar killing but at a detectably lower efficiency. In each case, the ∆*tssM* mutant has completely lost the ability to kill the coincubated bacteria (Fig. 1D). We also assessed the antifungal activity of Ab25 and found that it was able to kill *Saccharomyces cerevisiae*, *Candida albicans,* and *Candida glabrata* at similar efficiencies after 6 hours of coincubation (Fig. 1E). Thus, the T6SS of Ab25 is capable of targeting both Gram-negative, Gram-positive, and eukaryotic microorganisms.

### The T6SS of strain Ab25 deploys three effectors for its antimicrobial activity

**Fig 2:**
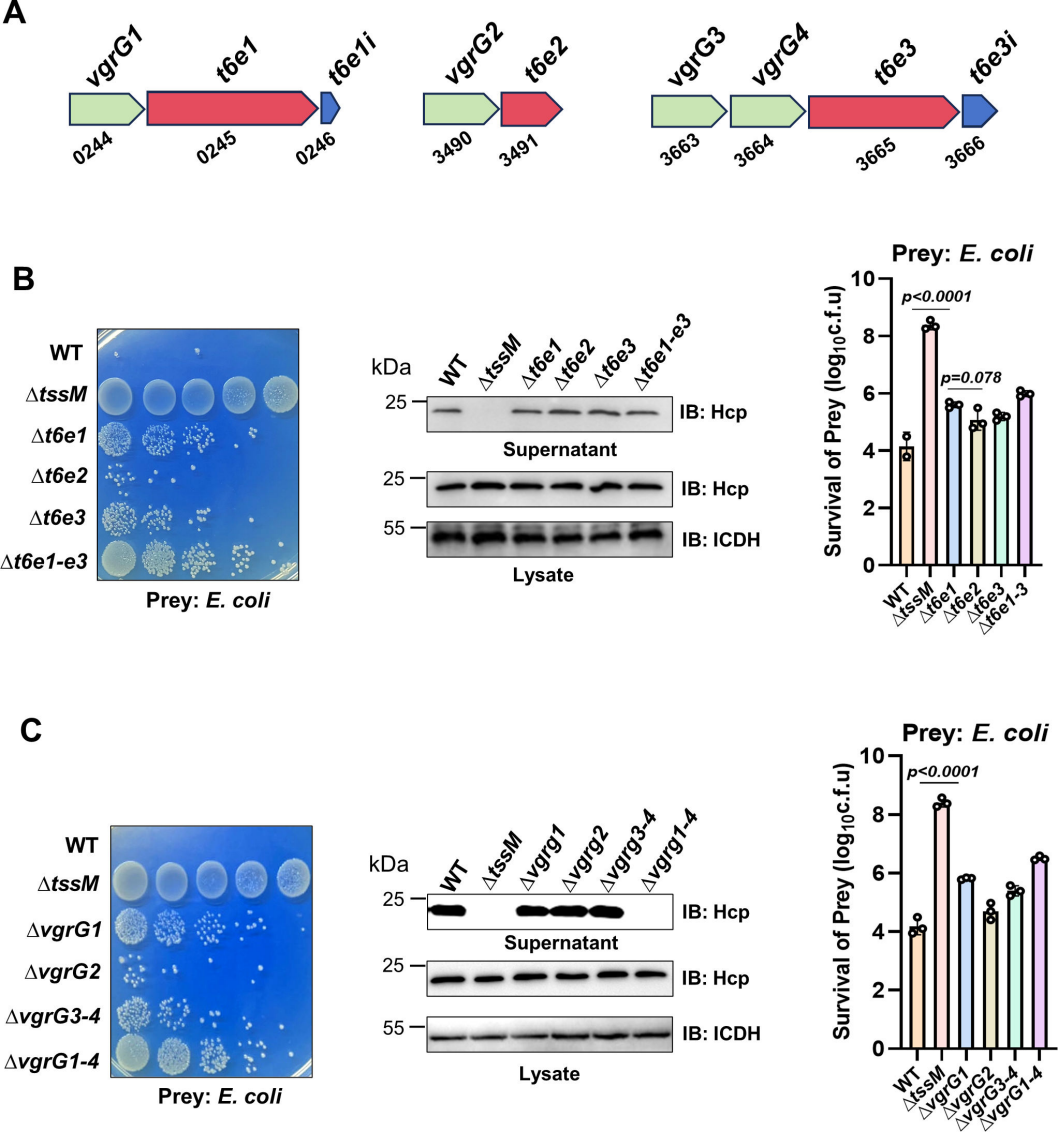
Ab25 expresses three predicted effectors that contribute differently to its killing ability (**A)** A diagram of the genetic organization of the genes for three effectors, corresponding VgrG proteins, and potential immunity proteins. Note that two predicted *vgrG* genes are proceeding the effector *t6e3*. (**B)** The role of the three effectors in killing bacteria by strain Ab25. Cells of wild-type and mutants lacking the indicated genes, where each was mixed with *E. coli* cells at a 1:1 ratio. The survival of *E. coli* cells was determined by spotting serially diluted cells onto selective medium. Images of a representative experiment are shown (left). The secretion of Hcp by these strains was determined by immunoblotting. ICDH was probed as a loading control (middle). Survival rates (mean ± SE) of *E. coli* cells from three independent experiments are shown (right panel). (**C)** The role of the predicted *vgrG* genes in the antibacterial activity of Ab25. Cells of the indicated mutants were mixed with *E. coli,* as described in panel B, to assess their killing ability. The secretion of Hcp by these strains was determined (middle), and the survival rates (mean ± SE) of the prey bacteria (left) were similarly determined. Note that an effector mutant displayed similar killing ability to that of the mutant lacking the corresponding *vgrG* gene.

In many cases, genes coding for T6SS effectors and *vgrG* are in close proximity (39). Sequence analysis identified four predicted VgrG proteins in the genome of Ab25 (Fig. 2A). Among these, *vgrG1*, *vgrG2,* and *vgrG4* each is upstream of the gene predicted to code for an effector, and the *vgrG3* gene is immediately upstream of *vgrG4* (Fig. 2A).

We examined the role of putative effectors by constructing mutants that lacked each of these genes. When assayed for antimicrobial activity, deletion of *t6e1* caused the most severe defects in killing *E. coli*, displaying a significant reduction comparing to the wild-type strain. The killing ability of the ∆*t6e3* mutant was detectably higher than that of ∆*t6e1*. In contrast, deletion of *t6e2* only slightly affected the ability of Ab25 to kill *E. coli* (Fig. 2B). Interestingly, a mutant lacking all three effector genes still retained considerable killing ability (Fig. 2B), suggesting the existence of additional effectors important for its competition against other microbial species.

We also examined the impact of *vgrG* genes on the killing ability of Ab25. In agreement of the notion that target cell delivery of the effector requires the cognate VgrG protein, the defect in killing displayed by strain ∆*vgrG1* was similar to that of the *t6e1* mutant and to that of the ∆*vgrG2* and ∆*t6e2* mutants (Fig. 2C). Deletion of both *vgrG3* and *vgrG4* resulted in a mutant that was defective in killing ability, similar to the ∆*t6e3* strain (Fig. 2C). While deletion of all three effector genes did not affect the secretion of Hcp, deletion of all four *vgrG* rendered the bacterium disabled the firing of the T6SS (Fig. 2B and C).

### A short carboxyl domain of T6e1 with DNase activity is sufficient for killing target microbes

**Fig 3:**
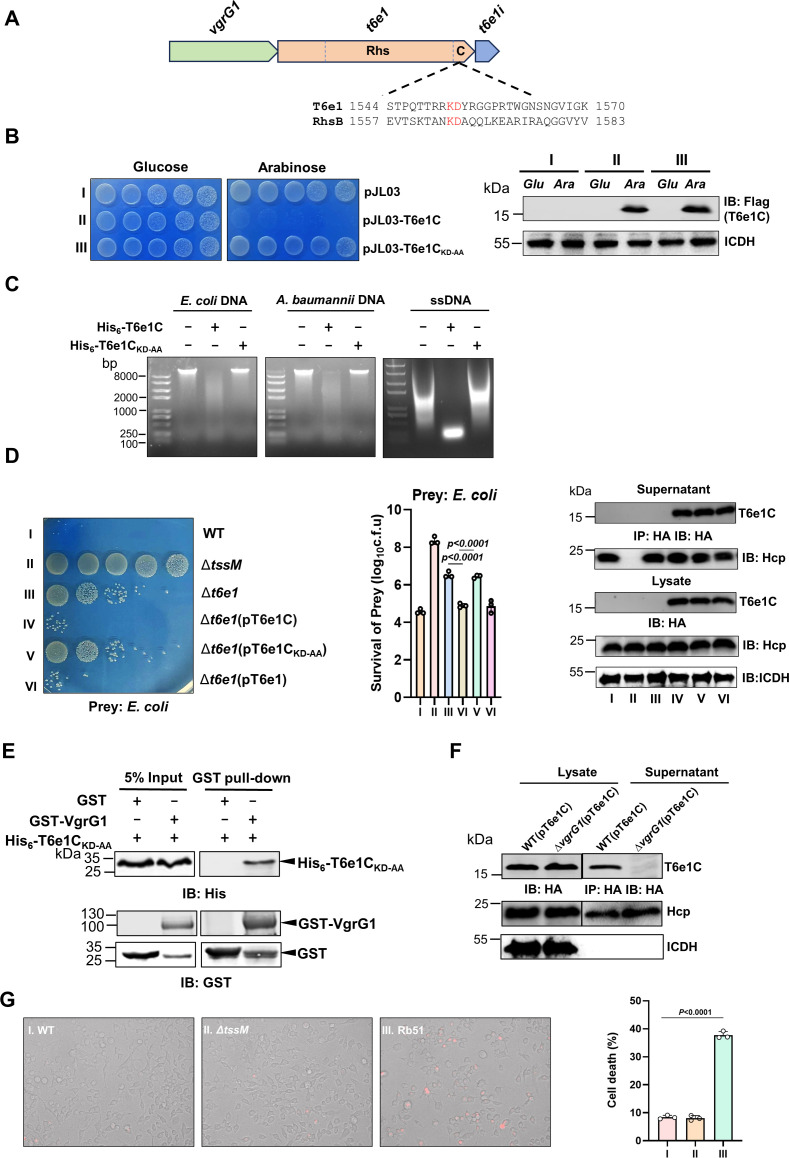
The effector T6e1 important for antimicrobial activity is a DNase (**A)** Gene organization of *t6e1* and its cognate *vgrG* and immunity genes. The two amino acids predicted to be important for the toxicity of T6e1 were in red letters in the blowout of its carboxyl end. RhsB from *A. citrulli* (29) was used as the reference protein. (**B)** The 163-residue fragment of the carboxyl end of T6e1 exerts its bacterial toxicity. The fragment or its derivative with mutations in the two residues predicted to be important for its activity was expressed from the arabinose-inducible promoter in *E. coli*. Serially diluted cells (fivefold) were spotted onto the medium with or without arabinose. Images were acquired after incubation at 37°C for 18 hours (left). The expression of the proteins was detected by immunoblotting with the Flag-specific antibody (right). (**C)** T6e1 has DNase activity. Recombinant T6e1 or T6e1_KD-AA_ was incubated with the genomic DNA of *E. coli*, *A. baumannii,* or single-stranded DNA from salmon sperm at 37°C for 1 hour. The integrity of DNA was evaluated by agarose gel electrophoresis and ethidium bromide staining. (**D)** The carboxyl terminal 163 residue of T6e1C is sufficient to complement the defect of the Δ*t6e1* mutant in bacterial killing. Cells of the indicated *A. nosocomialis* strains were mixed with an equal number of *E. coli* cells for 4 hours, and survival of the prey bacteria was determined by spotting serial dilutions onto selective medium (left). The rates of survival were quantitated (middle) (mean ± SE) from three independent experiments, each done in triplicate. The expression and secretion of Flag-T6e1C were determined by immunoblotting of the total cell lysates and culture supernatant, respectively. (**E and F)** VgrG1 interacts with T6e1 and is required for its secretion. GST-VgrG1 and His_6_-T6e1C_KD-AA_ were mixed, and the potential protein complex was captured using GST beads (upper panels). Protein input in reactions was detected by withdrawing 5% samples before adding GST beads (**E**) HA-T6e1C was expressed in wild-type or the ∆*vgrG1* mutant, and its secretion was determined by probing the culture supernatant by immunoblotting with the HA-specific antibody. (**G)** Strain Ab25 does not kill mammalian cells. Wild-type, the ∆*tssM* mutant, or the *B. abortus* vaccine strain RB51 was used to infect RAW 264.7 cells at an MOI of 200 for 4 hours, and cells were stained with ethidium bromide. Images were acquired using an X-83 Olympus fluorescence microscope. Quantitative results (mean ± SE) (right) were obtained from three independent experiments, each done in triplicate.

Among the three effector proteins, T6e1 and T6e3 are members of the rearrangement hotspot (Rhs) family proteins, which are composed of an amino terminal domain of unknown function, an RHS core, and a carboxyl terminal (CT) domain containing highly variable toxin motifs (40) (Fig. 3A). Because T6e1 has the greatest contribution to the killing ability of strain Ab25 among the three effectors, we further analyzed its mechanism of action. We first examined the role of its CT domain by expressing a fragment of 163 residues (T6e1C) from the arabinose-inducible promoter P_BAD_ (41, 42). On LB agar containing 2% glucose, an *E. coli* strain harboring the plasmid grew comparably to the strain harboring the empty vector. Induction of expression by arabinose completely abolished the ability of this *E. coli* strain to grow (Fig. 3B). Importantly, mutations in K1552 and D1553 (T6e1C_KD-AA_), the two residues predicted to be critical for the toxicity of Rhs toxins (40) in this fragment, abolished its ability to kill bacteria without impacting its stability (Fig. 3B).

The CT domains of Rhs members have been shown to have a range of activities, including DNase and RNase activities (43, 44). To determine the activity of T6e1, we purified recombinant His_6_-Sumo-T6e1C and His_6-_sumo-T6e1C_KD-AA_ from *E. coli* and assayed their ability to degrade DNA from various sources. Incubation of genomic DNA from *E. coli*, *A. baumannii,* or single-strand DNA from salmon sperm with His_6_-Sumo-T6e1C for 60 minutes led to complete degradation. In contrast, the mutant His_6-_sumo-T6e1C_KD-AA_ had completely lost the ability to eliminate DNA (Fig. 3C). Interestingly, when ssDNA was used as the substrate, the degradation product migrated at approximately the 250 bp position, which is similar to the observation in an earlier study using a different nuclease effector (38). Whether these nucleases target different forms of DNA differently remains to be investigated.

Introduction of a plasmid that directs the expression of T6e1C into the Δ*t6e1* mutant almost fully restored its ability to kill prey bacteria. Although expressed and secreted at similar levels, the strain complemented to express the enzymatically inactive mutant T6e1C_KD-AA_ exhibited a defect similar to that of the Δ*t6e1* mutant (Fig. 3D). Thus, the CT domain of 163 residues of T6e1 can be recognized by the T6SS for its delivery into target cells, and its DNase activity is necessary and sufficient for its role in killing prey microorganisms.

The ability of *t6e1C* to complement the Δ*t6e1* mutant suggests that this active domain of the effector can still be recognized by the T6SS, likely via an interaction with VgrG1. Indeed, when GST-VgrG1 and His_6_-T6e1C_KD-AA_ were mixed, T6e1C_KD-AA_ can be captured together with GST-VgrG1 by GST beads (Fig. 3E). In agreement with this observation, HA-T6e1C is secreted into the culture supernatant by the T6SS in a VgrG1-dependent manner (Fig. 3F).

The observation that Ab25 can effectively kill thick-walled fungi and the fact that T6e1 indiscriminately degrades DNA of different sources prompted us to examine whether this bacterium can kill mammalian cells. To this end, we tested its ability to induce cell death on RAW 264.7 cells. Infection of this macrophage with wild-type Ab25 or its ∆*tssM* mutant at a multiplicity of infection (MOI) of 200 did not detectably cause cell death (Fig. 3G). As a control, infections with the *Brucella abortus* vaccine strain RB51 known to induce cell apoptosis (45) at the same MOI caused extensive cell death (Fig. 3G).

### The activity of T6e1 is inhibited by an immunity protein

**Fig 4:**
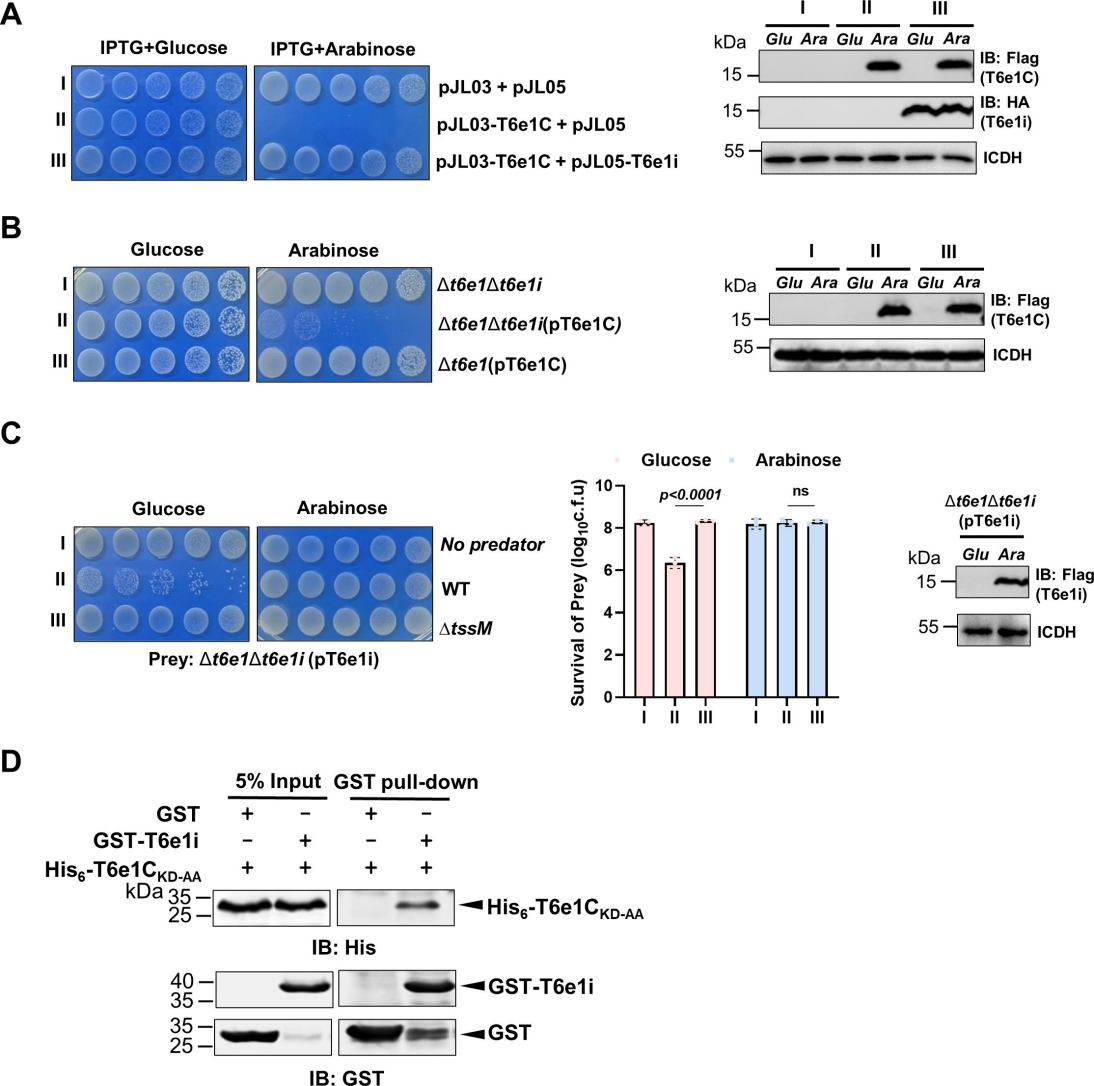
An immunity protein inhibits the activity of T6e1 by direct interactions (**A)** A plasmid that allows arabinose-inducible expression of T6e1C was co-transformed with one that harbors IPTG-inducible expression of T6e1i or an empty vector. A strain harboring two empty vectors was included as an additional control. Serially diluted bacterial cells were spotted onto the medium containing the indicated inducers. Images were acquired 18 hours after incubation at 37°C. The expression of the proteins was probed by immunoblotting, and ICDH was detected as a loading control. Similar results were obtained in at least three independent experiments. (**B)** The immunity protein protects *A. nosocomialis* from being killed by T6e1C. Flag-T6e1C expressed from the arabinose-inducible promoter was transformed into the Δ*t6e1*Δ*t6e1i* and the Δ*t6e1* mutant. The viability of the bacterial strains was assessed by spotting serially diluted cells onto plates with or without arabinose. Images were acquired after incubation at 37°C for 18 hours (left). The expression of Flag-T6e1C was assessed by immunoblotting (right). (**C)** T6e1i protects *A. nosocomialis* from being attacked by itself. Cells of wild-type and the Δ*tssM* mutant mixed the Δ*t6e1*Δ*t6e1i* mutant at 37°C for 4 hours on glucose and arabinose plates, respectively. Serially diluted cells were spotted onto selective medium with or without arabinose. Images were acquired after incubation at 37°C for 18 hours (left). Quantitative results (mean ± SE) (middle) were from three independent experiments, each done in triplicate. The expression of T6e1i was detected by immunoblotting with the Flag-specific antibody (right). (**D)** T6e1C directly interacts with T6e1i. GST or GST-T6e1i protein was mixed with His_6_-T6e1C_KD-AA_ and subjected to GST pull-down analysis. Co-captured proteins were probed by immunoblotting.

To avoid self-killing by toxic effectors for interbacterial competition, the activity of many toxins is inhibited by immunity proteins, which are often encoded by genes adjacent to the effector genes (46, 47). Sequence analysis revealed the presence of a small gene capable of coding a protein of 131 residues of no predicable function downstream of *t6e1* (Fig. 2A). To test whether this protein can confer immunity to the toxicity of T6e1, we introduced a plasmid that constitutively expressed the gene into an *E. coli* strain that expressed T6e1C from the arabinose-inducible promoter. While induction of T6e1C alone completely arrested bacterial growth, coexpression of the putative immunity protein and the toxic fragment rescued the growth of the bacteria (Fig. 4A). Thus, this small open-reading frame immediately downstream of *t6e1* codes for an immunity protein against the toxic effector. We designated this immunity gene as *t6e1i*.

Similarly, expression of T6e1C in the *A. nosocomialis* mutant Δ*t6e1*Δ*t6e1i* effectively killed the bacterial cells. In contrast, the Δ*t6e1* mutant that still expressed T6e1i can tolerate T6e1C (Fig. 4B). Furthermore, the Δ*t6e1*Δ*t6e1i* mutant can be killed effectively by wild-type Ab25, and such killing was prevented when T6e1i was expressed in the mutant (Fig. 4C). Together, these results indicate that T6e1i prevents the bacterium from being killed by its cognate toxic effector.

We next examined the mechanisms of inhibition of T6e1 by T6e1i by determining the interactions between these two proteins. Because the immunity protein effectively inhibited the toxicity of T6e1C, we incubated recombinant GST-T6e1i with His_6_-T6e1C_KD-AA_. We used the mutant because it is technically less challenging to prepare in high-quality protein due to its loss of toxicity. Interactions between these two proteins were determined by capturing the potential protein complex with agarose beads coated with glutathione. His_6_-T6e1C_KD-AA_ was co-purified with GST-T6e1i by GST beads (Fig. 4D), suggesting that T6e1i inhibits the toxicity of T6e1 by direct interactions via recognition of the region close to its carboxyl terminus with DNase activity.

### T6e1 is processed into three fragments by self-cleavage

**Fig 5:**
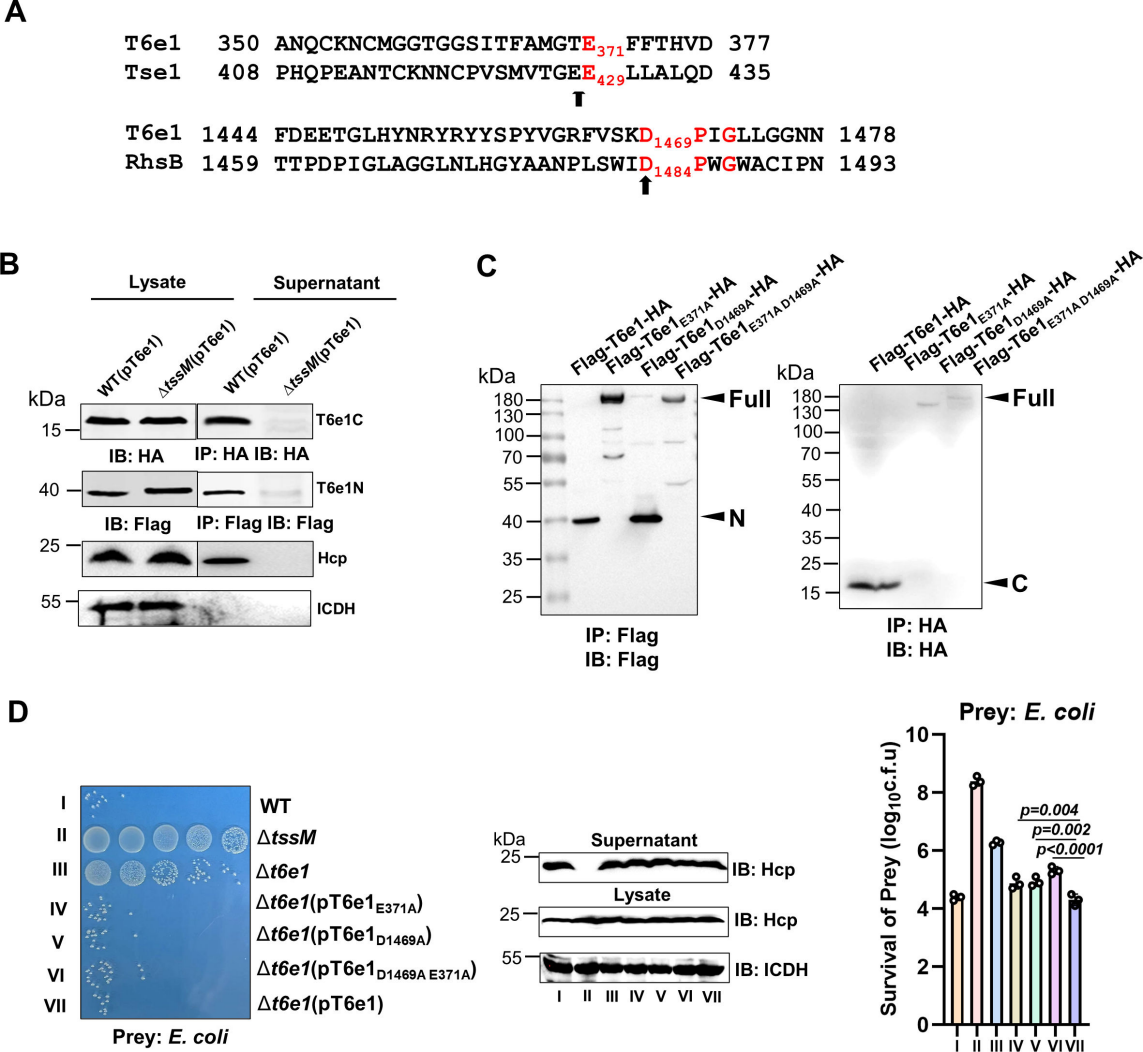
T6e1 undergoes self-cleavage at two positions (**A)** Predicted cleavage sites in the amino and carboxyl regions of T6e1 identified by aligning the sequences of T6e1 and RhsB. (**B)** T6e1 expression and secretion. Plasmids expressing Flag-T6e1-HA were separately introduced into the wild-type and the Δ*tssM* mutant, and the expression of T6e1 was probed by immunoblotting. The culture supernatant was subjected to immunoprecipitation to determine protein secretion. (**C)** Detection of T6e1 and its cleavage-defective mutants. Bacterial strains expressing the indicated alleles of *t6e1* were induced with 2% arabinose, and total lysates were subjected to immunoprecipitation. Proteins of interest in the precipitates were detected with the antibody specific to the epitope. (**D)** Complementation of the ∆*t6e1* mutant with alleles of *t6e1* defective in self-cleavage. The indicated *A. nosocomialis* strains were co-incubated with *E. coli* for 4 hours, and the killing activity was assessed by determining viable cells of *E. coli*. Representative images (left) of one experiment are shown. Quantitative results (mean ± SE) (right) were from three independent experiments, each done in triplicate. The expression and secretion of Hcp were determined by immunoblotting with ICDH as a loading control.

Some members of the RHS family effectors of the T6SS are known to undergo self-cleavage (48, 49). To test whether similar events occurred in T6e1, we expressed Flag-T6e1-HA in strain Δ*t6e1*. The protein products of this construct were probed by immunoblotting following immunoprecipitation with antibody specific for Flag or HA. Despite multiple attempts, we were unable to detect full-length Flag-T6e1-HA using either antibody. Instead, a protein of approximately 17 kDa was detected in total bacterial lysates when Flag-T6e1-HA was expressed in both the wild-type strain or the T6SS-defective mutant ∆*tssM*. This signal was also detected in the culture supernatant of the wild-type but not the ∆*tssM* mutant, suggesting that T6e1 undergoes cleavage and the CT domain is secreted by the T6SS (Fig. 5B). A protein of about 40 kDa was detected with the Flag-specific antibody that recognized the epitope in the amino terminus of the fusion protein. Interestingly, this fragment is also secreted into the culture supernatant by the T6SS (Fig. 5B).

Sequence inspection identified E371 and D1469, two conserved residues known to be the cleavage sites for relevant members of the Rhs protein family (49). To determine whether these positions are indeed the cleavage sites, we created three substitution mutants: T6e1_E371A_, T6e1_D1469A,_ and T6e1_E371AD1469A_. Plasmids that direct the expression of these mutants were introduced into Ab25, and the expression of these alleles was examined. Probing with the Flag antibody detected a protein close to full-length Flag-T6e1_E371A_-HA, which was not detectable using the HA antibody (Fig. 5C), suggesting that the D371A mutation has abolished the cleavage occurring at the amino terminus portion. Similarly, the Flag antibody allowed the detection of a protein of about 40 kDa, which corresponds to T6e1_1-371_ in cells expressing the Flag-T6e1_D1469A_-HA mutant. When this sample was probed with the HA antibody, a protein of approximately 140 KDa was detected, which was corresponding to T6e1_1-1469_ (Fig. 5C). Finally, full-length T6e1 was detected in cells expressing the Flag-T6e1_E371AD1469A_-HA mutant (Fig. 5C). These results establish that T6e1 undergoes two events of self-cleavage at E371 and D1469.

To investigate how self-cleavage impacts the bactericidal activity of T6e1, we complemented the Δ*t6e1* strain with the cleavage-defective mutants T6e1_E371A_, T6e1_D1469A,_ or T6e1_E371A D1469A_ and examined the ability of the resulting strains to kill *E. coli*. While expression of T6e1 fully restored the killing ability of the Δ*t6e1* mutant, complementation by each of the three cleavage-deficient mutants exhibited lower killing ability (Fig. 5D).

### Overexpression of VgrG4 inhibits T6SS firing in strain Ab25

**Fig 6:**
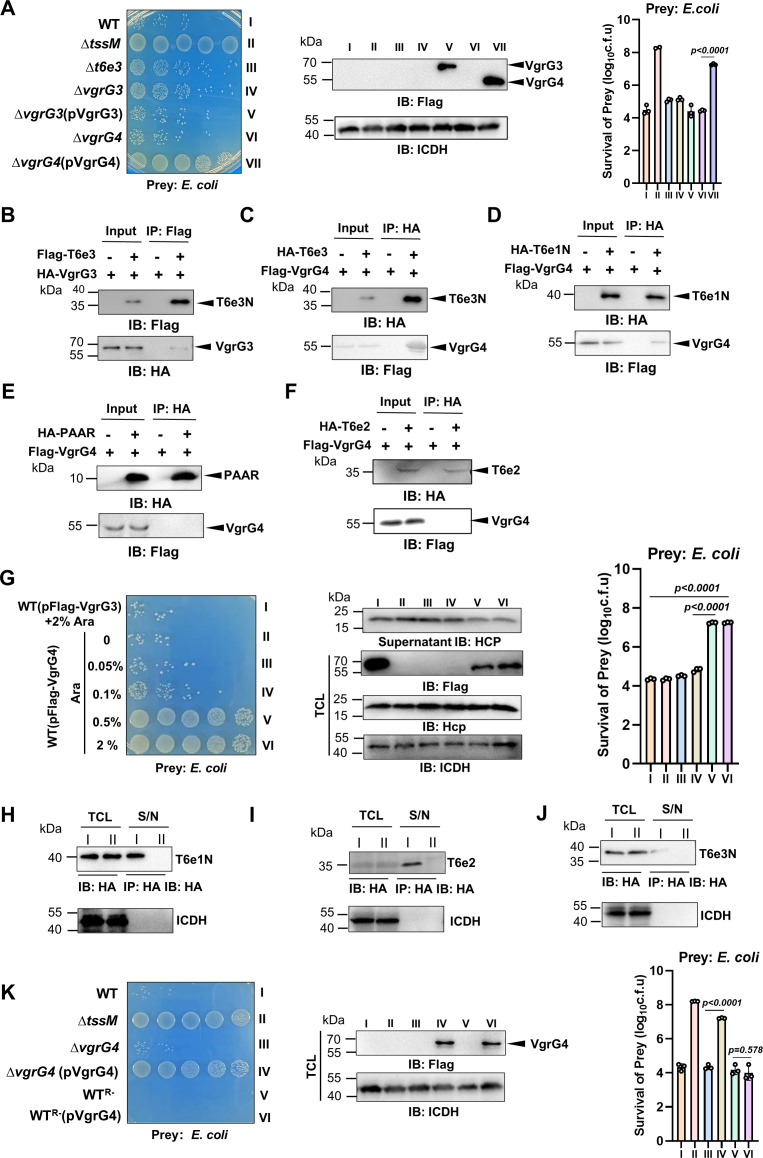
Overexpression of VgrG4 inhibits the firing of the T6SS in strain A25 (**A)** Expression of VgrG4 from a plasmid inhibits the antibacterial activity of the ∆*vgrG4* mutant. Cells of the indicated strains derived from Ab25 were mixed with *E. coli* cells, and the survival of the latter was determined. Representative images of the spotted cells (left) and quantitative results (mean ± SE) from three independent experiments, each done in triplicate (right), are shown. The expression of Flag-VgrG3 and Flag-VgrG4 in relevant strains was probed by immunoblotting, ICDH was probed as a loading control (middle). (**B–F)** VgrG4 interacts with a subset of T6SS effectors. Lysates of cells expressed the indicated protein pairs were subjected to immunoprecipitation with antibody specific for the indicated epitope tag. Protein interactions were determined by detecting the presence of potential binding proteins in the precipitates by immunoblotting. The binding between VgrG3 and T6e3 was included as a control (**B**). Note that VgrG4 interacts with T6e3N (**C**) and T6e1N (**D**) but not PAAR (**E**) or T6e2 (**F and G)** VgrG4 inhibits the bacterial killing ability of Ab25 in a dose-dependent manner. Cells of strain Ab25 harboring Flag-VgrG4 expressed from the P_BAD_ promoter was induced by the indicated concentrations of arabinose that were mixed with *E. coli* cells for 4 hours prior to determining viable prey cells. A strain expressing VgrG3 was included as a control. Representative images of the spotted cells (left) and quantitative results (mean ± SE) from three independent experiments, each done in triplicate, (right) are shown. The secretion of Hcp and expression of VgrG by the strain differently induced by arabinose were also determined (middle). Note that high-level expression of VgrG4 blocks the secretion of Hcp without impacting its expression. (**H–J)** Overexpression of VgrG4 inhibits effector secretion. Secretion of effector Te61 (**H**), T6e2 (**I**), and T6e3 (**J**) was determined by probing their presence in the culture supernatant by immunoblotting. ICDH in both total lysates and culture supernatant was detected as controls. I, the bacterial strain that expressed only the effector; II, the strain that expressed both the effector and Flag-VgrG4. (**K)** VgrG4-mediated inhibition of T6SS firing is specific to strain Ab25. Cells of the indicated strains and of *E. coli* were mixed for 4 hours, and the survival of the latter was determined. Representative images of the spotted cells (left) and quantitative results (mean ± SE) from three independent experiments, each done in triplicate (right), are shown. The expression of Flag-VgrG4 was probed (middle). Note that overexpression of VgrG in strain WT^R-^, which was derived from strain ATCC17978, did not detectably affect its antibacterial activity.

Differing from T6SSs in which a VgrG protein often pairs with its cognate effector, the *t6e3* gene of strain Ab25 is adjacent to two *vgrG* genes (Fig. 2A). To determine which VgrG is involved in the injection of T6e3, we first compared the killing ability of the ∆*t6e3*, ∆*vgrG3,* and ∆*vgrG4* mutants. The ∆*t6e3* and ∆*vgrG3* mutants displayed similar reduction in killing *E. coli*. Deletion of *vgrG4* also affected the killing ability but at a detectably lower level (Fig. 6A). These results suggest that VgrG3 functions together with T6e3. Indeed, we found that T6e3 interacted with VgrG3 (Fig. 6B).

We also constructed complementation strains of ∆*vgrG3* and ∆*vgrG4* by introducing plasmids expressing the corresponding gene into these mutants. As expected, although not to the level of wild-type bacteria, expression of VgrG3 restored the killing ability of strain ∆*vgrG3*. Intriguingly, expression of VgrG4 almost completely abolished the antimicrobial activity of the ∆*vgrG4* mutant, and the reduction in killing ability was close to that displayed by the ∆*tssM* mutant with a defective T6SS (Fig. 6A). To further probe the mechanism of inhibition, we examined the interactions between VgrG4 and other effector and PAAR, which revealed that VgrG4 interacted with T6e3 and T6e1 but not with T6e2 or PAAR (Fig. 6C through F).

To more carefully examine the inhibitory effects, we introduced a plasmid that allowed arabinose-inducible expression of VgrG4 into wild-type Ab25 and assessed its killing ability after incubation with different amounts of the inducer. Incubation with wild-type Ab25 killed most of the prey bacteria, whereas the killing ability was slightly decreased when the expression of VgrG4 was induced with 0.05% or 0.1% arabinose for 4 hours. Notably, Flag-VgrG4 was barely detectable by immunoblotting under these conditions. When the concentration of arabinose was increased to 0.5% or 2%, the killing ability of Ab25 was significantly decreased, which was close to that seen in experiments involving the ∆*tssM* mutant (Fig. 6G). The inhibitory effect was specific to VgrG4 because similarly expressed VgrG3 did not detectably impact the killing ability of Ab25 (Fig. 6G).

We also examined the secretion of Hcp, an essential component of the T6SS that is delivered into the extracellular milieu upon VgrG4 overexpression (50–52). Although overexpression of VgrG4 does not impact the total protein level of Hcp, its secretion was blocked in a dose-dependent manner (Fig. 6G, middle panel). Overexpression of VgrG4 also inhibited the secretion of T6e1, T6e2, and T6e3 (Fig. 6H through J), which was consistent with its inhibitory effect on Hcp secretion. We also examined the strain specificity of VgrG4 by introducing the plasmid into strain WT^R-^, a pAB3-cured strain derived from ATCC17978 that constitutively expresses its T6SS (38). The killing ability of this strain was not affected by VgrG4 overexpression (Fig. 6K). Thus, inhibition of T6SS firing by VgrG4 is specific to strain Ab25.

### The T6SS promotes the survival of Ab25 in niches with prey bacteria

**Fig 7:**
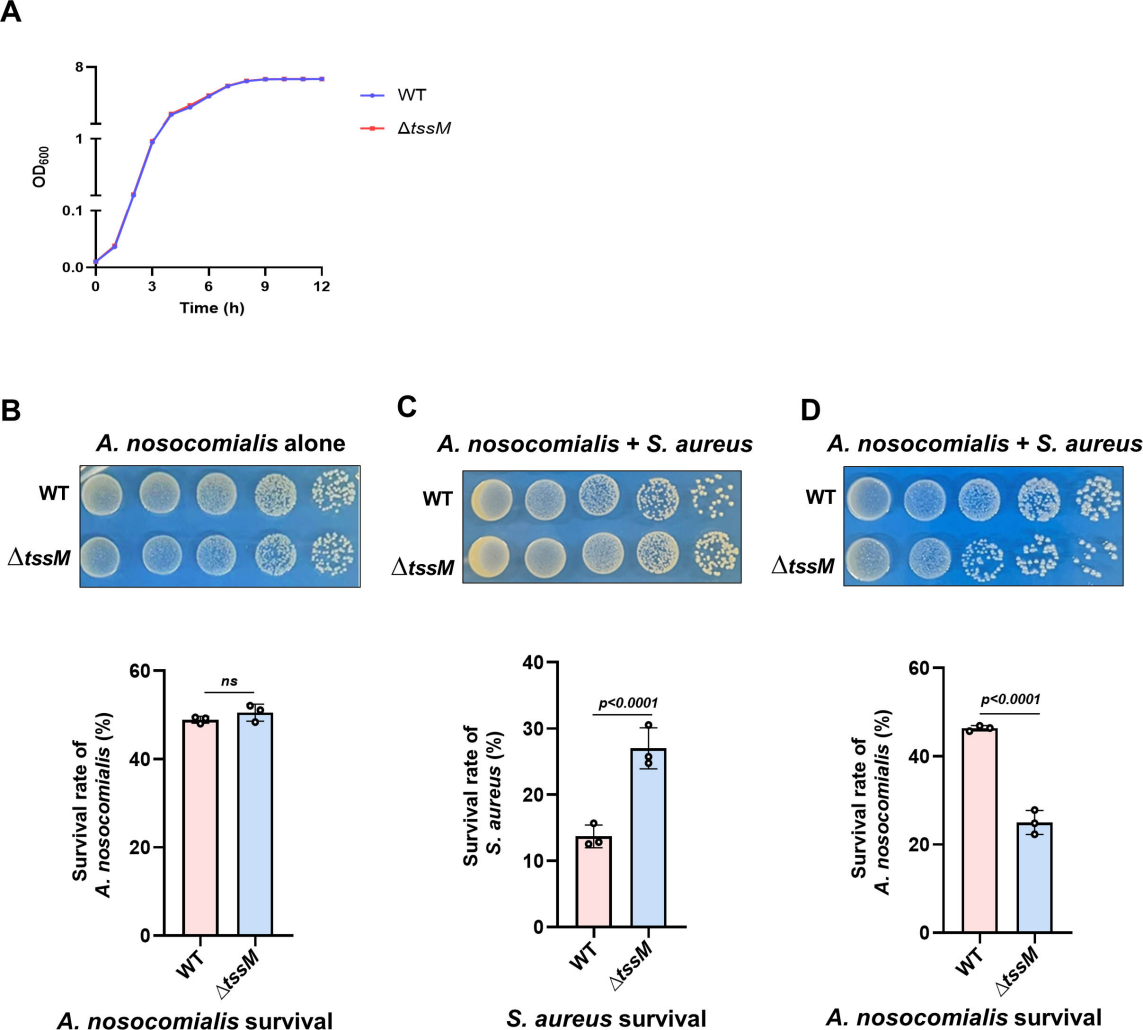
The T6SS promotes long-time bacterial survival in niches with prey microbes (**A)** The T6SS is not required for strain Ab25 to grow in the bacteriological medium. Overnight cultures of strain Ab25 or its ∆*tssM* mutant were diluted into fresh LB broth at a 1:100 dilution, and the growth of the bacteria was monitored by measuring OD_600_ at 3-h intervals. (**B–D)** The T6SS promotes A. *nosocomialis* survival on the dry surface with co-existing bacteria. Cells of wild-type Ab25 or the ∆*tssM* mutant defective in the T6SS were spotted onto the plastic surface alone (**B**) or together with *S. aureus* cells at a 1:1 ratio (**C and D**). The survival of *A. nosocomialis* and *S. aureus* was determined at 1-day intervals for 10 days. Results shown are representative images of the spotted bacterial cells at the tenth day timepoint. Quantitative results (mean ± SE) from three independent experiments, each done in triplicate at the tenth day, are shown (lower portion). Note that the T6SS allows *A. nosocomialis* to survive better at the cost of *S. aureus*. The survival rate of *A. nosocomialis* after 10 days of coexistence with *S. aureus* on simulated plastic medical devices.

Bacteria of the *Acinetobacter* genus can survive for extended periods on dry surfaces of medical equipment, which often is the source of nosocomial infections (53). Although T6SSs endow bacteria with the ability to kill or inhibit the growth of competing microorganisms (54, 55), its role in survival on dry surfaces is less studied. To explore this function, we first examined the growth of Ab25 and the ∆*tssM* mutant in the bacteriological medium and found that these two strains grew indistinguishably (Fig. 7A), indicating that the T6SS does not play a role in bacterial replication in ample nutrient supply. Next, we mixed cells of Ab25 or the Δ*tssM* mutant with *S. aureus* on the surface of dry plastic materials. The survival of the two bacterial strains was assessed daily for a period of 15 days. For samples in which only cells of Ab25 or the ∆*tssM* mutant were spotted, and viable cells of the wild-type strain and the Δ*tssM* mutant were indistinguishable at all timepoints examined (Fig. 7B), suggesting that the function of the T6SS is not important for its survival when the bacteria were incubated on the surface alone. Importantly, we found that after incubation for 10 days, viable cells of *S. aureus* mixed with wild-type Ab25 became significantly lower than those that had been mixed with the Δ*tssM* mutant (Fig. 7C). Furthermore, the decrease in *S. aureus* cells corresponded well with better survival of wild-type Ab25, whose viable cells became significantly higher than that of the Δ*tssM* strain starting from the tenth day (Fig. 7D). Taken together, these results indicate that the T6SS endows a survival advantage to Ab25 in niches co-occupied by other bacterial species, probably due to nutrients released by killed cells.

## DISCUSSION

Much of the current knowledge about *Acinetobacter* spp., including their virulence, environmental adaptability, and resistance to antimicrobials, is obtained by studying *A. baumannii,* particularly the prototype strain ATCC17978 (56, 57). The expression of the type VI secretion system essential for interbacterial competition in strain ATCC17978 is repressed by two TetR-like proteins encoded by the plasmid pAB3 (36). Such repression is believed to allow better spread of the conjugative pAB3 (36, 38). By screening a collection of clinical isolates of *Acinetobacter* spp., we found that strain Ab25 has potent antimicrobial activity. Genes coding for components of the T6SS in Ab25 are conserved and syntenic to many *A. baumannii* strains (58) (Fig. S1; Table S1).

Sequence analysis of the regions flanking the T6SS locus in the genome of strain Ab25 did not identify any canonical mobile genetic elements such as transposons, integrons, or insertion sequences that are typically indicative of horizontal gene transfer. Interestingly, the regions adjacent to the T6SS are rich in transport systems and regulatory proteins, including multiple ABC transporter components and transcriptional regulators from the LysR family. The presence of these elements suggests a complex regulatory network that may facilitate expression or mobilization of the T6SS under specific environmental conditions. It is possible that the T6SS was integrated into the genome of *Acinetobacter* spp. in the distant past, and any signs of direct transfer, such as flanking direct repeats or associated transposases, might have been lost or diverged beyond recognition over time.

In contrast to high-level conservation in its structural genes, effectors of the T6SS in *Acinetobacter* spp. vary greatly in protein size, enzymatic activity, and distribution on the chromosome (59). Based on the number of VgrG proteins, strain Ab25 is predicted to harbor three effectors, among which T6e1 appears to play the most important role in its antimicrobial activity. In contrast, deletion of T6e2 only caused a marginal reduction in its ability to kill prey bacteria (Fig. 2A and B). Interestingly, while the activity of T6e1 is inhibited by an immunity protein (Fig. 4), T6e2 is not accompanied by an immunity protein (Fig. 2A). The physiological role of less toxic effectors such as T6e2 needs further investigation. One possibility is that such effectors may target cells that contain molecules capable of enhancing their destruction potency.

The DNase effector T6e1 has a few salient features. First, it belongs to the conserved Rhs-family proteins (40) with a size of 1,570 residues, which is similar to RhsB from *Acidovorax citrulli* (29) and TseI from *Aeromonas dhakensis* (49). Secretion of RhsB requires both a cognate VgrG protein and the chaperone EagT2 coded for by an adjacent gene (29). T6e1 has a cognate VgrG protein but not a chaperone (Fig. 2A). Second, a carboxyl region of 163 residues is sufficient to kill target cells when expressed in the ∆*t6e1* mutant (Fig. 3D). Consistently, VgrG1 interacted with this domain of T6e1 and was required for its secretion (Fig. 3E and F). In the absence of a chaperone required for optimal secretion, the role of the remaining approximately 90% of the protein is unknown. Like RhsB and TseI (29, 49), T6e1 also undergoes two events of self-cleavage, and the abolishment of such cleavage only slightly impacted its antimicrobial activity (Fig. 5). Since T6e1C, a fragment of the last 163 amino acids of T6e1, retained the ability to almost fully restore the defect of killing by the ∆*t6e1* mutant, it is likely that both full-length and cleaved products are delivered into target cells. Because protein synthesis is a metabolically demanding process (60), the advantage of synthesis of large effectors such as T6e1, RhsB, and TseI is not clear. This notion is even more apparent in light of the fact that much shorter truncation mutants of these large proteins are almost equally effective in killing competitive microbes.

Despite its ability to kill fungal cells, Ab25 does not detectably impact the viability of mammalian cells (Fig. 3G). Similarly, an earlier study found that the nuclease T6SS effector TefE does not exert toxicity to mammalian cells. Ectopic expression indicates that TafE can enter the nuclei of yeast but not mammalian cell nuclei (38). We speculate that the lack of effective nucleus localization signals in effectors such as T6e1 accounts for the inability of Ab51 to kill mammalian cells.

VgrG proteins are a part of the puncturing device of the T6SSs that are delivered into target cells. In addition to functioning as the spear of the injector (61), these proteins facilitate effector delivery by functioning as the carrier of cargo effectors (62), and they can also directly modulate the activity of target cells, particularly when fused to an additional domain of specific activity (63, 64). A VgrG protein from *A. baumannii* has been shown to target eukaryotic cells (65). One unique feature of the T6SS of strain Ab25 is the presence of two VgrG genes immediately upstream of *t6e3* (Fig. 2A). VgrG4 is not the carrier protein for T6e3, despite the fact that their genes are next to each other on the chromosome. In contrast, VgrG4 appears to negatively modulate the firing of the T6SS by strain Ab25 because overexpression of this protein strongly inhibited the secretion of Hcp and of all of its effectors (Fig. 6G through J).

Regulation of the T6SS at both transcriptional and posttranslational levels has been documented (66). For example, orthologs of the serine/threonine kinase PpkA, the cognate phosphatase PppA, and the forkhead-associated (FHA) domain–containing proteins regulate T6SS activity in *Agrobacterium tumefaciens* (67, 68)and *Pseudomonas aeruginosa* by distinct mechanisms (69). The observation that VgrG4 interacted with both T6e1 and T6e3 suggests that it may displace the cognate VgrG of these effectors when expressed at high levels. We propose a model in which VgrG4 regulates T6SS firing in strain Ab25 by modulating the formation of effector–VgrG complexes on the tip of the nanomachine. The inability of VgrG4 to inhibit the T6SS in strain ATCC17978 is likely due to the structurally different effectors found in these two strains.

T6SS has long been proposed to promote colonization by eliminating competitive microbes at given niches (70). Our results demonstrate that the T6SS of strain Ab25 allows the bacterium to better survive for a long term when coexisting with prey bacteria in environments with scarce nutrient supply (Fig. 7). The fact that such survival is at the cost of the co-existing microbes suggests that killed target cells provide the nutrients. *A. nosocomialis* has been shown to cause infections as severe as *A. baumannii* (71). The role of the T6SS in bacterial survival suggests that compounds capable of inactivating this secretion system will be useful in efforts aiming at eliminating these bacteria in niches such as dry surfaces of medical devices and relevant equipment.

## MATERIALS AND METHODS

### Bacterial strains and plasmid construction

The strains, plasmids, and primers used in the study are listed in Table S2. *A. nosocomialis* and *E. coli* were cultured in the LB medium. When needed, antibiotics were used at the following concentrations: kanamycin 30 µg/mL, gentamicin 10 µg/mL, and streptomycin 100 µg/mL. *A. nosocomialis* mutants were constructed following an established method (37). For complementation experiments, the genes of interest and their substitution mutants were cloned into appropriate plasmids. The sequences of primers used in this study are in Table S2.

### Genome sequencing and annotation

Genomic DNA was extracted using the standard SDS method (72), verified for integrity via agarose gel electrophoresis, and quantified using a Qubit 2.0 Fluorometer (Thermo Scientific). Library construction for the PacBio Sequel platform involved using the SMRT Bell Template kit 1.0 (Pacific Biosciences), focusing on generating long-read libraries with an insert size of approximately 10 kb, followed by size selection using the BluePippin System (Sage Science). Concurrently, Illumina sequencing libraries were prepared using the NEBNext Ultra DNA Library Prep Kit for Illumina (New England Biolabs, USA) starting from 1 µg of DNA per sample, fragmented by sonication to approximately 350 bp. These libraries underwent end-repair, A-tailing, adapter ligation, and were amplified via PCR, with the final products purified and quantified using an Agilent 2100 Bioanalyzer (Agilent Technologies).

The genome assembly process was initiated by filtering reads under 500 bp to ensure high-quality data input. Long reads (over 6,000 bp) from the PacBio Sequel system were assembled using the SMRT Link v5.0.1 software (Pacific Biosciences), employing its integrated error correction algorithms to enhance the accuracy of the assembly. The preliminary assembly was further refined using the Arrow algorithm within the SMRT Link software to correct base-level errors and improve consensus sequence quality. Subsequently, Illumina short reads were aligned to the draft assembly using BWA-MEM (73), which facilitated the identification and correction of potential misassembly and provided additional depth for variant detection.

Annotation was performed using a combination of automated pipelines and manual curation to ensure comprehensive coverage of genomic features. Protein-coding genes were predicted using Prokka (74), which integrates tools such as Prodigal (75) for open-reading frame prediction. Functional annotation was enriched by querying against multiple databases including GO, KEGG, COG, and SWISS-Prot using BLASTp (76) with an e-value cutoff of 1e−5. Noncoding RNAs were identified using tRNAscan-SE (77) for tRNA genes and RNAmmer (78) for rRNA genes.

### Bacterial and fungal killing assays

To determine interspecies killing, testing strains of *A. nosocomialis* were mixed with the *E. coli* [strain DH5α(pET28a)], *E. cloacae,* (38) or *S. aureus* (38). Briefly, bacterial cells from overnight cultures grown to saturation were washed three times with PBS, and their OD_600_ was measured to estimate cell density. Cells of *A. nosocomialis* and *E. coli*, *E. cloacae,* or *S. aureus* were mixed in a 1:1 ratio. Twenty microliters of the bacterial solution was spotted onto LB agar without antibiotic selection, and the plates were incubated at 37°C for 4 hours. Collected bacteria were resuspended in PBS, and serial dilutions were spotted onto appropriate selection LB agar. Bacterial CFU was determined after incubation at 37°C for 18 hours.

To assess the ability to kill fungi, cells testing strains of *A. nosocomialis* were similarly mixed with cells of *S. cerevisiae* strain W303 (79), *C. albicans* (38), or *C. glabrata* (38) were mixed at a ratio of 10:1 on LB agar. After incubation at 30°C for 6 hours, cells were serially diluted and plated onto YPD plates containing 30 µg/mL of kanamycin. After 3 days, the survival of the prey cells was observed.

### Immunoblotting

To detect protein, bacterial cells corresponding to 1.0 OD_600_ collected by centrifugation were resuspended in 50 µL of SDS sample buffer, cells were lysed by boiling for 5 minutes, and 10 µL of the soluble faction was separated by SDS-PAGE. Protein was transferred to nitrocellulose membranes, blocked with 5% nonfat milk, and then incubated with specific antibodies. The information of antibodies used in this study is in Table S3. Signals were detected and quantitated using an Odyssey Calyx Imaging System (Li-Cor).

### Protein expression and purification

Plasmids for the expression of the protein of interest as GST- or His_6_-SUMO-tagged proteins were transformed into the *E. coli* strain BL21(DE3). Twenty milliliters of saturated bacterial culture grown in LB broth containing the appropriate antibiotics was added to 1 L fresh medium, and the culture was grown to OD_600_ = 0.6–0.8. Expression of the target protein was induced by adding isopropyl β-D-1-thiogalactopyranoside (IPTG) to a final concentration of 200 µM. After incubation for 18 hours at 16°C, cells were collected and lysed using a high-pressure homogenizer (JNBIO, Guangzhou, China). The soluble fraction of the lysates was incubated with 1 mL Ni^2+^-NTA (Qiagen, Cat# 30250) at 4°C for 1 hour. The resin was loaded into a column, and unbound proteins were removed by washing thrice, each with 10× beads volume of a washing buffer (50 mM NaH_2_PO_4_, 300 mM Nalco, and 20 mM imidazole). The target protein was eluted with 5 mL of the same buffer containing 250 mM imidazole. For GST-tagged proteins, GST resin was used, and the protein was eluted with PBS containing 10 mM glutathione. After measuring the concentration with the Bradford assay, proteins were dialyzed and then stored in the storage buffer (50 mM Tris-HCl, 150 mM NaCl, and 10% glycerol) at 4°C.

### Protein toxicity assay

To test the toxicity of the protein, the coding region of the testing genes and their mutant each was inserted into pJL03, which expresses protein from the arabinose-inducible P_BAD_ promoter (41). The gene for the immunity protein T6e1i against T6e1 was inserted into pJL05 for expression from the IPTG-inducible P_TAC_ promoter (37).

*E.coli* strains derived from DH5α carrying the indicated plasmids were cultured overnight in LB medium containing the appropriate antibiotics and 2% glucose. Collected cells resuspended in fresh LB broth containing 2% glucose were grown to OD_600_ of 0.8. After fivefold serial dilutions, 20 µL of the cells was spotted onto LB agar containing the appropriate inducers (glucose, arabinose, IPTG, or the indicated combinations). The bacterial growth (viability) was examined after incubation at 37°C for 18 hours.

### DNase activity assays

Bacterial genomic DNA was extracted using a Kit from TIANGEN (Cat# DP302-02), and salmon sperm DNA was obtained from Solarbio Technology Co., Ltd (Beijing, China). In each case, 1 µg DNA was mixed with the indicated recombinant protein to a final volume of 50 µL in a reaction buffer (20 mM NaCl, 2 mM MgCl_2_, 10 mM Tris-HCl, and 0.2 mM dithiothreitol). This reaction was allowed to proceed for a duration of 60 minutes at 37°C prior to separation by agarose electrophoresis. After EtBr staining, DNA was visualized using the Tanon imaging system.

### GST pull-down assay

Fifty micrograms of purified GST or GST-tagged protein was incubated with equal amounts of His_6_-T6e1C_KD-AA_ at 4°C for 4 hours. The potential protein complex was captured by adding 50 µL of GST beads to the reactions and incubating for 1 hour. The beads were washed five times with a washing buffer (500 mM NaCl in PBS), and bound proteins were solubilized with 50 µL SDS buffer prior to SDS-PAGE and subsequent detection by immunoblotting with the appropriate antibodies.

### Immunoprecipitation

Plasmids expressing Flag-T6e1-HA under the control of the P_BAD_ promoter were transformed into the wild-type, Δ*tssM,* or Δ*vgrG* mutant. Overnight cultures of the resulting bacterial strains were diluted 1:50 into 20 mL fresh LB broth, the cultures were grown until their OD_600_ reached 2. Expression of the protein was induced by adding arabinose to a final concentration of 2% for 4 hours at 37°C. Cells collected were lysed to obtain the soluble fraction by centrifugation at 10,000 × *g* for 15 minutes. The soluble supernatant was divided into two identical samples and into which 50 µL Flag or HA antibody beads were added. After incubation at 4°C for 14 hours, the beads were washed 3 times with PBS. Proteins solubilized with 40 µL sample were boiled for 5 minutes and were separated by SDS-PAGE prior to detection by immunoblotting with the appropriate antibodies.

### Bacterial survival on dry surface assays

Testing strains of *A. nosocomialis* and *S. aureus* were cultured in LB broth for 14 hours. Cells were suspended in PBS after washing thrice with PBS, and their density was adjusted by measuring the value of OD_600_. Twenty microliters of bacteria mixed at the indicated ratios was spotted onto plastic materials, and the samples were placed at room temperature with (moisture 40%–50%). *A. nosocomialis* cells not mixed with other bacteria were similarly treated and spotted. The survival of the bacteria was examined by determining the CFU of the testing strains for 15 days at 24-hour intervals.

### Bioinformatics and statistical analysis

Homologous sequences were aligned by the BLAST CD-search program (https://www.ncbi.nlm.nih.gov/Structure/cdd/wrpsb.cgi). The gene sequences of the components of the T6SS aligned with *A. baumannii* 17978 were performed by the HHpred program (80) and the BLAST CD-search program. Genome sequence map and comparative genome circles were generated by BRIG software (81). Quantitative data were processed and analyzed using GraphPad Prism 9 software, and a two-tailed Student’s *t*-test was employed for significance analysis.

## Data Availability

All data described in this paper are available in the associated figures and supplemental tables and figures. The genome sequence of strain Ab25 has been deposited at the GenBank with an open accession number of CP154856.

## SUPPLEMENTAL MATERIAL

Fig. S1 mbio.01468-24-s0001.TIF Identification of an *Acinetobacter* isolate with potent antibacterial activity.

Fig. S2 mbio.01468-24-s0002.TIF The killing of *S. cerevisiae* and *S. aureus* by strain Ab25.

Fig. S3 mbio.01468-24-s0003.TIF Survival of of strain Ab25 or its Δ*tssM* mutant defective in T6SS.

Legends mbio.01468-24-s0004.docx Legends for supplemental figures.

Table S1 mbio.01468-24-s0005.pdf SPredicted T6SS components of strain Ab25 and their similarity to those of strain ATCC17978.

Table S2 mbio.01468-24-s0006.pdf Bacterial strains, plasmids, and primers used in this study.

Table S3 mbio.01468-24-s0007.pdf Antibodies used in this study.

ASM does not own the copyrights to Supplemental Material that may be linked to, or accessed through, an article. The authors have granted ASM a non-exclusive, world-wide license to publish the Supplemental Material files. Please contact the corresponding author directly for reuse.
