# Supplementary figures and images for: Acinetobacter nosocomialis utilizes a unique type VI secretion system to promote its survival in niches with prey bacteria
Source: mBio. 2024 Jun 25;15(7):e01468-24. doi: 10.1128/mbio.01468-24 (PMC11253628; doi:10.1128/mbio.01468-24)

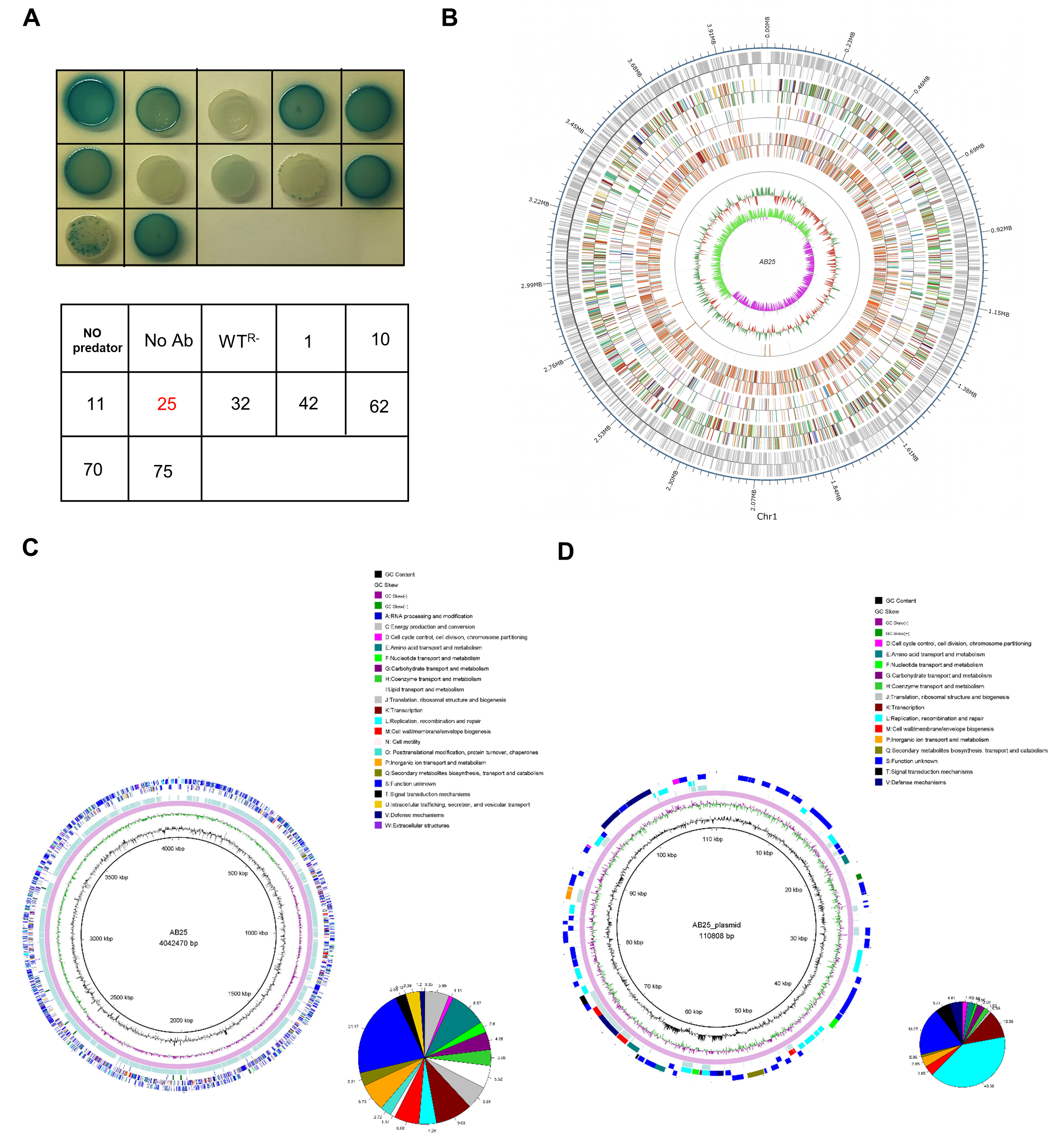

Supplement: Fig. S1 — Identification of an Acinetobacter isolate with potent antibacterial activity. [file mbio.01468-24-s0001.tif]

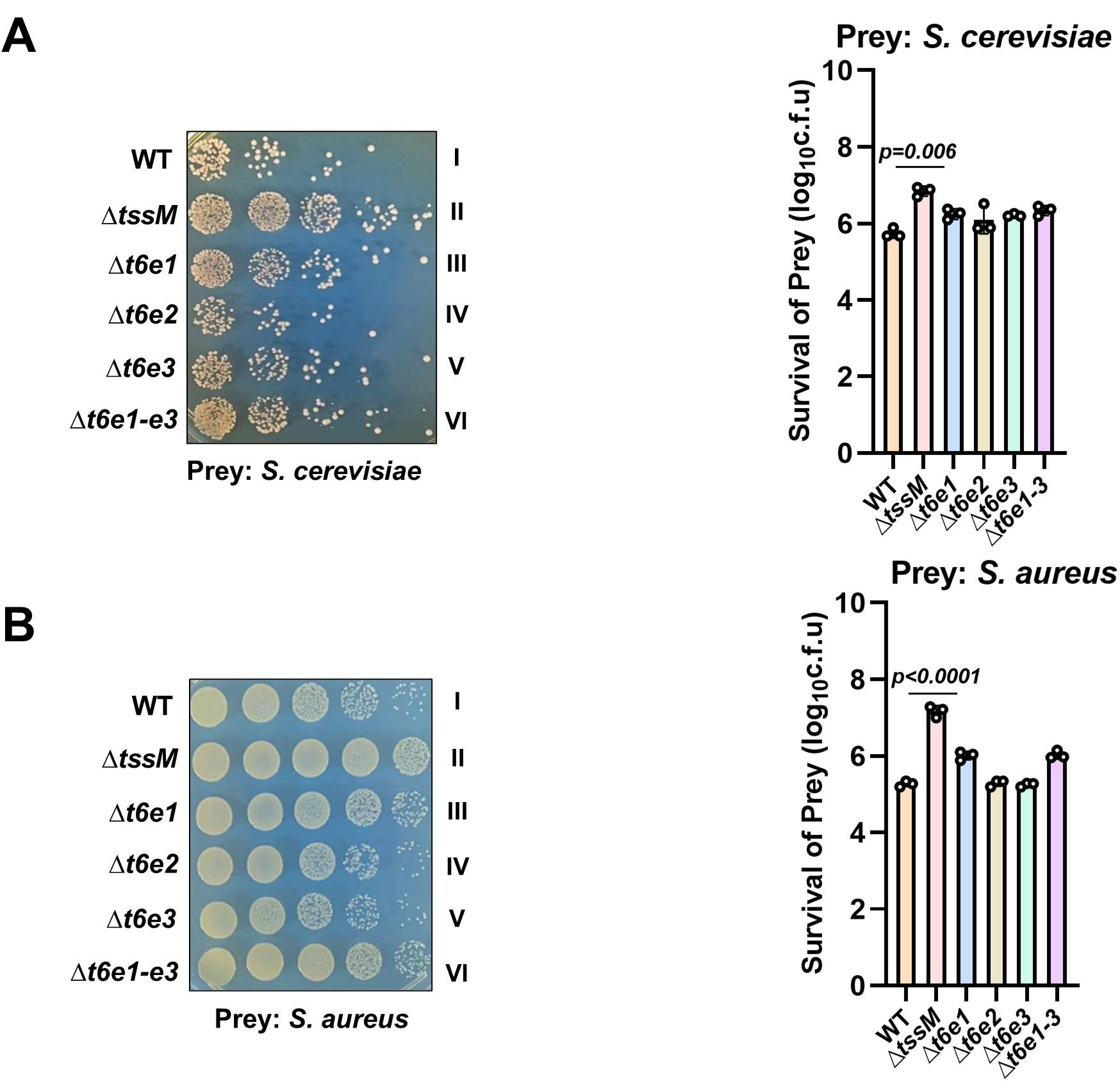

Supplement: Fig. S2 — The killing of S. cerevisiae and S. aureus by strain Ab25. [file mbio.01468-24-s0002.tif]

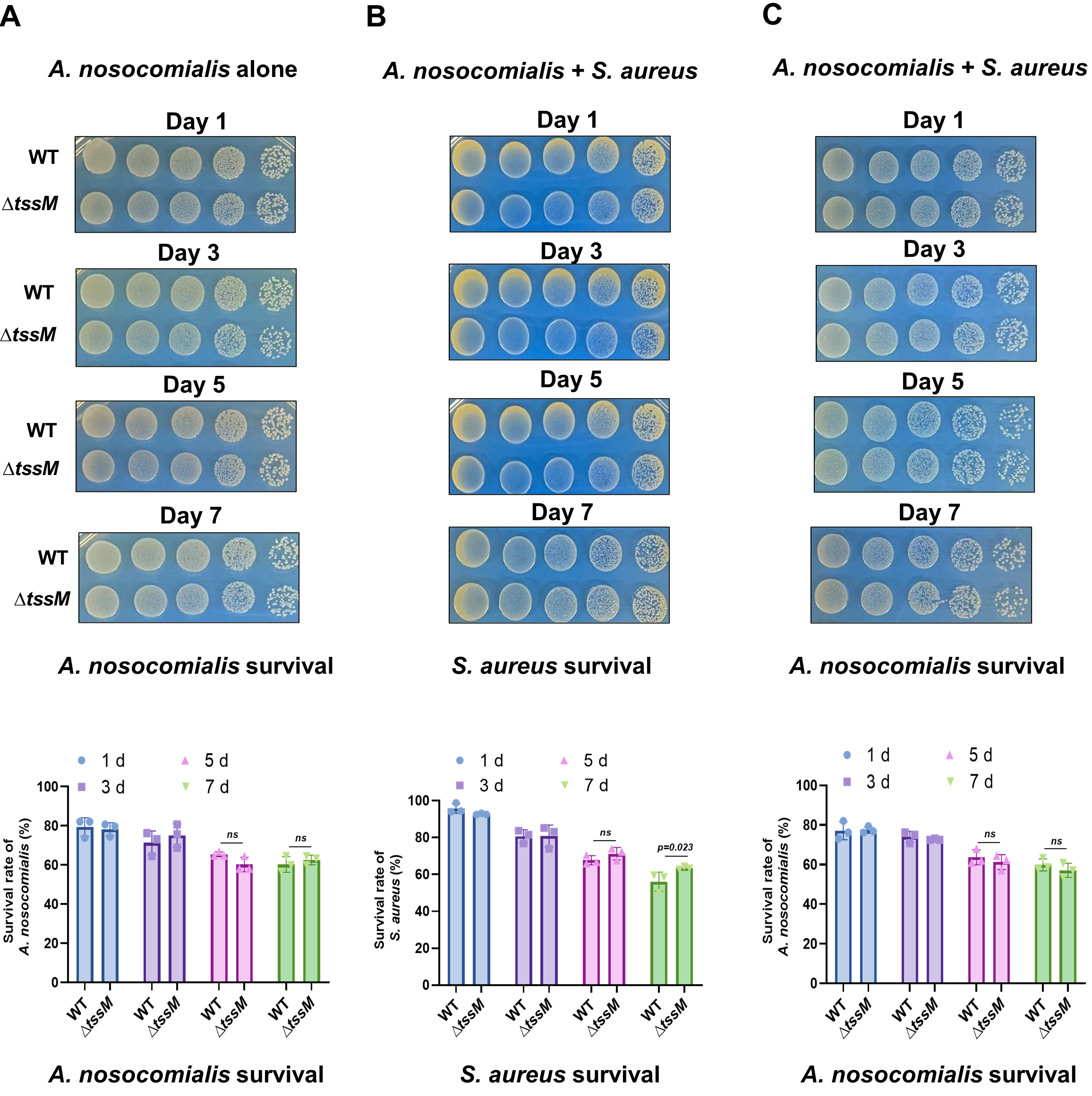

Supplement: Fig. S3 — Survival of of strain Ab25 or its ΔtssM mutant defective in T6SS. [file mbio.01468-24-s0003.tif]
