# Supplementary material for: Acinetobacter nosocomialis utilizes a unique type VI secretion system to promote its survival in niches with prey bacteria
Source: mBio. 2024 Jun 25;15(7):e01468-24. doi: 10.1128/mbio.01468-24 (PMC11253628; doi:10.1128/mbio.01468-24)
Supplement: Legends — for supplemental figures. [file mbio.01468-24-s0004.docx]

**Legends of Supplementary Figures**

**Fig. S1** Identification of an *Acinetobacter* isolate with potent antibacterial activity

**A.** Cells of a panel of *Acinetobacter* isolates were mixed with cells of an *E.* *coli* strain expressing β-galactosidase for 4 h. Samples were spotted onto LB agar containing X-gal. The killing ability of *Acinetobacter* isolates was evaluated by the formation of blue bacterial lawn. Acb, *A. baumannii.*

**B-D.** Genome sequence of strain Ab25. The map of the chromosome and a plasmid of 110 kbp (B). Comparative genome circles of AB25 chromosome with AB17978 chromosome and AB25 plasmid with AB17978 chromosome (C,D) respectively. The map was generated by BRIG software.

**Fig. S2** The killing of *S. cerevisiae* and *S. aureus* by strain Ab25. Cells of strain Ab25 were mixed with *S. cerevisiae* (A) or *S. aureus* (B) at 10：1 ratio for 6 h. The survival of the prey cells was evaluated by determining the number of viable cells. Results shown were representative images of the spotted bacterial cells (left panels). Quantitative results (mean ± s.e.) from 3 independent experiments each done in triplicate were shown (right panels).

**Fig. S3** Cells of strain Ab25 or its ∆*tssM* mutant defective in T6SS were spotted onto plastic surface alone (A) or together with *S. aureus* cells at 1:1 ratio (B and C). The survival of *A. nosocomialis* and *S. aureus* on the 1^st^ , 3^rd^, 5^th^ and the 7^th^ day were shown. Quantitative results (mean ± s.e.) from 3 independent experiments each done in triplicate at the indicated time were shown (lower panels).
