# Supplementary material for: Acinetobacter nosocomialis utilizes a unique type VI secretion system to promote its survival in niches with prey bacteria
Source: mBio. 2024 Jun 25;15(7):e01468-24. doi: 10.1128/mbio.01468-24 (PMC11253628; doi:10.1128/mbio.01468-24)
Supplement: Table S1 — SPredicted T6SS components of strain Ab25 and their similarity to those of strain ATCC17978. [file mbio.01468-24-s0005.pdf]

Table S1 Predicted T6SS components of strain Ab25 and their similarity to those of strain ATCC17978

Gene code	Length (aa)	Corresponding T6SS proteins	E-value
0308	168	*tssB*	$4e^{-81}$
0309	494	*tssC*	0
0310	168	*hcp*	$5e^{-130}$
0311	159	*tssE*	$1e^{-119}$
0312	846	*tssF*	$6e^{-164}$
0313	333	*tssG*	0
0315	1280	*tssM*	0
0316	320	*tagF*	0
0317	256	*tagN*	0
0318	88	PAAR	$5e^{-64}$
0319	894	*tssH*	0
0320	365	*tssA*	0
0321	455	*tssK*	0
0322	269	*tssL*	0
