# Supplementary material for: Acinetobacter nosocomialis utilizes a unique type VI secretion system to promote its survival in niches with prey bacteria
Source: mBio. 2024 Jun 25;15(7):e01468-24. doi: 10.1128/mbio.01468-24 (PMC11253628; doi:10.1128/mbio.01468-24)
Supplement: Table S2 — Bacterial strains, plasmids, and primers used in this study. [file mbio.01468-24-s0006.pdf]

Table S2 Bacterial strains, plasmids and primers used in this study

Bacterial Strains	Source	Identifier
Ab25	Clinical isolate	N/A
Ab25Δ*tssM*	This study	N/A
Ab25Δ*tssB*	This study	N/A
Ab25Δ*tssC*	This study	N/A
Ab25Δ*tssF*	This study	N/A
Ab25Δ*hcp*	This study	N/A
Ab25Δ*tagF*	This study	N/A
Ab25Δ*vgrG1*	This study	N/A
Ab25Δ*vgrG2*	This study	N/A
Ab25Δ*vgrG3-4*	This study	N/A
Ab25Δ*vgrG1-4*	This study	N/A
Ab25Δ*t6e1*	This study	N/A
Ab25Δ*t6e2*	This study	N/A
Ab25Δ*t6e3*	This study	N/A
Ab25Δ*t6e1-3*	This study	N/A
Ab25Δ*t6e1-t6e1i*	This study	N/A
Ab25(pJL03)	This study	N/A
Ab25Δ*tssM*(pJL03)	This study	N/A
Ab25Δ*t6e1*(pJL03)	This study	N/A
Ab25Δ*t6e1*(pJL03::*t6e1C*)	This study	N/A
Ab25Δ*t6e1*(pJL03::$t6e1C_{KD\text{-}AA}$)	This study	N/A
Ab25(pJL03::*t6e1C*)	This study	N/A
Ab25Δ*tssM*(pJL03::*t6e1C*)	This study	N/A
Ab25Δ*vgrG1*(pJL03::*t6e1C*)	This study	N/A
Ab25Δ*t6e1-t6e1i*(pJL03::*t6e1C*)	This study	N/A
Ab25Δ*t6e1-t6e1i*(pJL03::*t6e1i*)	This study	N/A
Ab25Δ*t6e1*(pJL03::*t6e1*)	This study	N/A
Ab25Δ*t6e1*(pJL03::$t6e1_{D1469A}$)	This study	N/A
Ab25Δ*t6e1*(pJL03::$t6e1_{E371A}$)	This study	N/A
Ab25Δ*t6e1*(pJL03::$t6e1_{E371A\ D1469A}$)	This study	N/A
E. cloacae (Clinical isolate)	This study	N/A
S. aureus (Clinical isolate)	This study	N/A
C. albicans (Clinical isolate)	This study	N/A
C. glabrata (Clinical isolate)	This study	N/A
S. cerevisiae(W303)	(*1*)	N/A
E. coli BL21(DE3)	TransGen	Cat# CD601
E.coli DH5a	(*2*)	Cat# KTSM101L

**Plasmids**	**Source**	**Identifier**
pJL03	(*3*)	N/A
pJL03::*t6e1*	This study	N/A
pJL03::*t6e1*$_{KD-AA}$	This study	N/A
pJL03::*t6e1*$_{D1469A}$	This study	N/A
pJL03::*t6e1*$_{E371A}$	This study	N/A
pJL03::*t6e1i*	This study	N/A
pGEX6p-1	Cytiva	Cat#28-9546-48
pGEX6p-1::*t6e1i*	This study	N/A
pGEX6p-1::*vgrG1*	This study	N/A
pET SUMO	ThermoFisher	Cat#K30001
pET SUMO::*t6e1*	This study	N/A
pET SUMO::*t6e1*$_{KD-AA}$	This study	N/A
pET SUMO::*t6e1*$_{D1469A}$	This study	N/A
pET SUMO::*t6e1C*	This study	N/A
pET SUMO::*t6e1C*$_{KD-AA}$	This study	N/A
pJL05	(*3*)	N/A
pJL05::*t6e1i*	This paper	N/A
pET28a	Invitrogen	N/A
pSR47s	(*4*)	N/A
pSR47s::Δ*tssM*	This paper	N/A
pSR47s::Δ*tssB*	This paper	N/A
pSR47s::Δ*tssC*	This paper	N/A
pSR47s::Δ*tssF*	This paper	N/A
pSR47s::Δ*hcp*	This paper	N/A
pSR47s::Δ*tagF*	This paper	N/A
pSR47s::Δ*t6e1*	This paper	N/A
pSR47s::Δ*t6e2*	This paper	N/A
pSR47s::Δ*t6e3*	This paper	N/A
pSR47s::Δ*vgrG1*	This paper	N/A
pSR47s::Δ*vgrG2*	This paper	N/A
pSR47s::Δ*vgrG3-4*	This paper	N/A
pSR47s::Δ*t6e1i*	This study	N/A

Primers	Sequence (Restriction enzyme sites are underlined)	Note
t6e1-F	cgcggatccatggcacaaaattcagtcg	*t6e1* 5’ BamHI
t6e1-R	acgcgtcgactcaatagcttttcccatctaag	*t6e1* 3’ SalI
t6e1C-F	ctgggatccatggatccgattggattattgggt	*t6e1C* 5’ BamHI
t6e1C-R	acgcgtcgactcaagcgtagtctgggacgtcgtatgggtaatagcttttcccatc	*t6e1C* 3’ SalI
t6e1i-F	cgcggatccatgaaaaataagctctggaaaa	*t6e1i* 5’ BamHI
t6e1i-R	acgcgtcgacttaattttcaaaaaaaatatattgatct	*t6e1i* 3’ SalI
vgrG1-F	ctgggatccatgttgaacagtattcatcaagttttagatagtttg	*vgrG1* 5’ BamHI
vgrG1-R	ctggtcgacttatacaaacgtttcaaaaggtgattggat	*vgrG1* 3’ SalI
t6e1$_{KD-AA}$-1	tcggccccctctataagctgctctgcgggtggtttgtg	*t6e1*$_{KD-AA}$ 5’
t6e1$_{KD-AA}$-2	cacaaaccacccgcagagcagcttatagagggggccga	*t6e1*$_{KD-AA}$ 3’
t6e1$_{D1469A}$-1	cccaataatccaatcggagctttacttacaaacctacctacata	*t6e1*$_{D1469A}$ 5’
t6e1$_{D1469A}$-2	tatgtaggtaggtttgtaagtaaagctccgattggattattggg	*t6e1*$_{D1469A}$ 3’
t6e1$_{E371A}$-1	cgacatgggtaaagaacgcggtacccattgcaaag	*t6e1*$_{E371A}$ 5’
t6e1$_{E371A}$-2	ctttgcaatgggtaccgcgttctttacccatgtcg	*t6e1*$_{E371A}$ 3’
t6e1-KO-A-F	ctggtcgacctcggctgatgcacaaat	*t6e1* A 5’*Sac*I
t6e1-KO-A-R	tcaaacctttttgtacaataaatggggactaaaagtatttaaag	*t6e1* A 3’
t6e1-KO-B-F	tgctcctttaaatacttttagtccccatttattgtacaaaaaagg	*t6e1* B 5’
t6e1-KO-B-R	ctggagctcatatttggaataaaacctattgatgcatattt	*t6e1* B 3’ *Sal*I
t6e2-KO-A-F	ctggtcgactgatattaaaggccagctaccg	*t6e2* A 5’*Sac*I
t6e2-KO-A-R	gttggaccatcacttcatccatagactggaatgttttaaaaaatg	*t6e2* A 3’
t6e2-KO-B-F	tcagacattttttaaaacattccagtctatggatgaagtgatggt	*t6e2* B 5’
t6e2-KO-B-R	ctggagctctttactcactaattaaaaagtgaaatatataccacc	*t6e2* B 3’ *Sal*I
t6e3-KO-A-F	ctggtcgacctaaaagcagtcgcagcac	*t6e3* A 5’*Sac*I
t6e3-KO-A-R	taatggttctgccttgcgcttcaacaggttgtttttaatttctt	*t6e3* A 3’
t6e3-KO-B-F	acataaagaaattaaaaaacaacctgttgaagcgcaaggcagaac	*t6e3* B 5’
t6e3-KO-B-R	ctggagctcactttcatttttcttcaaatggatatttattaga	*t6e3* B 3’ *Sal*I
t6e1i-KO-A-F	ctggtcgacagcggagtaaacatcaccatacag	*t6e1i* A 5’*Sac*I
t6e1i-KO-A-R	aaatccatatttaaaagattcagtttgaaatttatatttttcca	*t6e1i* A 3’
t6e1i-KO-B-F	tttcaaactgaatcttttaaatatggatttaaagatcaatatatt	*t6e1i* B 5’
t6e1i-KO-B-R	ctggagctccatttatattttaatttgtgatttaaagcatctgtacgagtc	*t6e1i* B 3’ *Sal*I
vgrG1-KO-A-F	ctggagctcctttagtactttctacacgtgc	*vgrG1* A 5’*Sac*I
vgrG1-KO-A-R	ggatttttgttattgctgttgctaatgcccaaactatctaa	*vgrG1* A 3’
vgrG1-KO-B-F	ttagatagtttgggcattagcaacagcaataacaaaaatcc	*vgrG1* B 5’
vgrG1-KO-B-R	acgcgtcgacattggcctttgtcgtttct	*vgrG1* B 3’ *Sal*I
vgrG2-KO-A-F	ctggagctcgtaactggcgcaatttttta	*vgrG2* A 5’*Sac*I
vgrG2-KO-A-R	aatgcacctttttgtgctgcagcaaaaccaattttatctaa	*vgrG2* A 3’
vgrG2-KO-B-F	ttagataaaattggttttgctgcagcacaaaaaggtgcatt	*vgrG2* B 5’
vgrG2-KO-B-R	acgcgtcgactctttataaatcaattttaaacaaataaaatt	*vgrG2* B 3’ *Sal*I
vgrG3-4-KO-A-F	ctggagctcacgacaccacttaaatcatc	*vgrG3-4* A 5’*Sac*I
vgrG3-4-KO-A-R	tctaaagccccttgctgaatgagaaaaccaacactttcta	*vgrG3-4* A 3’

vgrG3-4-KO-B-F	tagaaagtgttggttttctcattcagcaaggggctttaga	*vgrG3-4* B 5’
vgrG3-4-KO-B-R	acgcgtcgactaaagtgtctcctttcgtatgg	*vgrG3-4* B 3’ *Sal*I
tssM-KO-A-F	ctggagctcggatgaagagaatttcgaatac	*tssM* A 5’*Sac*I
tssM-KO-A-R	caggttgtgtaggattcgtgatgtactg	*tssM* A 3’
tssM-KO-B-F	cacgaatcctacacaacctgctgcaa	*tssM* B 5’
tssM-KO-B-R	acgcgtcgacttgttattcacccaaaaaagtc	*tssM* B 3’ *Sal*I
tssC-KO-A-F	ctggagctcaaaaaaatgatttattagctaaagcag	*tssC* A 5’*Sac*I
tssC-KO-A-R	aagtcgtaaagaaacttcattttca	*tssC* A 3’
tssC-KO-B-F	cttgttgaaaatgaagtttctttac	*tssC* B 5’
tssC-KO-B-R	ctggtcgaccataatgcgcccatattatgt	*tssC* B 3’ *Sal*I
tssB-KO-A-F	ctggagctcaaatttatagtaattttaattcttaataaattccat	*tssB* A 5’*Sac*I
tssB-KO-A-R	acgaatctgatcagttcgaattcgt	*tssB* A 3’
tssB-KO-B-F	cttcaacgaattcgaactgatcaga	*tssB* B 5’
tssB-KO-B-R	ctggtcgacatctgtaattgcaatttcagttgg	*tssB* B 3’ *Sal*I
tssF-KO-A-F	ctggagctctcaaattgaagcacgttctagtttct	*tssF* A 5’*Sac*I
tssF-KO-A-R	taacatgtttcatggctgtgaacgacagaaca	*tssF* A 3’
tssF-KO-B-F	ttcctgcttgttctgtcgttcacagccatgaa	*tssF* B 5’
tssF-KO-B-R	ctggtcgactctgcaataaggcagcttgtagca	*tssF* B 3’ *Sal*I
tagF-KO-A-F	ctggagctcgttggtttcagtgaaattgatcagc	*tagF* A 5’*Sac*I
tagF-KO-A-R	gtctaaatatttttaaaccttgattcggacttttcccata	*tagF* A 3’
tagF-KO-B-F	tgtattatgggaaaagtccgaatcaaggtttaaaa	*tagF* B 5’
tagF-KO-B-R	ctggtcgacataatgaatcatttactcttgctgctga	*tagF* B 3’ *Sal*I
hcp-KO-A-F	ctggagctcatttctcatgttgctgcagcagcg	*hcp* A 5’*Sac*I
hcp-KO-A-R	tattattagaaagtgaccatttcttatcaactttatattt	*hcp* A 3’
hcp-KO-B-F	gcggtaaatataaagttgataagaaatggtcactt	*hcp* B 5’
hcp-KO-B-R	ctggtcgacccacttaaatgaataggtagtaattttacatcttgagttg	*hcp* B 3’ *Sal*I

N/A, not applicable.

References

1. H. Y. Fan, K. K. Cheng, H. L. Klein, Mutations in the RNA polymerase II transcription machinery suppress the hyperrecombination mutant hpr1 delta of Saccharomyces cerevisiae. *Genetics* **142**, 749-759 (1996).
2. D. Hanahan, J. Jessee, F. R. Bloom, Plasmid transformation of Escherichia coli and other bacteria. *Methods Enzymol* **204**, 63-113 (1991).
3. J. Jie, X. Chu, D. Li, Z. Luo, A set of shuttle plasmids for gene expression in Acinetobacter baumannii. *PLoS One* **16**, e0246918 (2021).
4. Z. Q. Luo, R. R. Isberg, Multiple substrates of the Legionella pneumophila Dot/Icm system identified by interbacterial protein transfer. *Proc Natl Acad Sci U S A* **101**, 841-846 (2004).
