# Supplementary material for: Acinetobacter nosocomialis utilizes a unique type VI secretion system to promote its survival in niches with prey bacteria
Source: mBio. 2024 Jun 25;15(7):e01468-24. doi: 10.1128/mbio.01468-24 (PMC11253628; doi:10.1128/mbio.01468-24)
Supplement: Table S3 — Antibodies used in this study. [file mbio.01468-24-s0007.pdf]

Table S3 Antibodies used in this study

Antibody	Source or reference	Identifiers	Additional information
Rabbit polyclonal Anti-ICDH antibody	(1)	N/A	WB (1:3000)
Rabbit polyclonal Anti-HCP antibody	(1)	N/A	WB (1:3000)
Monoclonal Anti-Flag HRP antibody	Sigma	N/A	WB (1:3000)
Monoclonal Anti-HA HRP antibody	Abclonal	N/A	WB (1:1000)

References

(1) Luo J, Chu X, Jie J, Sun Y, Guan Q, Li D, Luo ZQ, Song L. 2023. Acinetobacter baumannii Kills Fungi via a Type VI DNase Effector. mBio 14:e0342022.
